# *Cordyceps farinosa* Cf-GZU06: Mycelium Culture Medium Optimization and Polysaccharide Characterization with Prebiotic Effects

**DOI:** 10.3390/foods15112038

**Published:** 2026-06-05

**Authors:** Yan-Chun Wang, Yu-Heng Mao, Meng-Qi Huang, Ting-Chi Wen, You Luo, Chun-Xiao Wang, Ang-Xin Song

**Affiliations:** 1 School of Liquor and Food Engineering, Guizhou University, Guiyang 550025, China; wangyanchun992@163.com (Y.-C.W.); 13765876890@163.com (M.-Q.H.); yluo9@gzu.edu.cn (Y.L.); cxwang@gzu.edu.cn (C.-X.W.); 2 School of Exercise and Health, Guangzhou Sport University, Guangzhou 510500, China; maoyh@gzsport.edu.cn; 3 The Engineering and Research Center for Southwest Bio-Pharmaceutical Resource of National Education Ministry of China, Guizhou University, Guiyang 550025, China; tingchiwen@yahoo.com

**Keywords:** *Cordyceps farinose*, liquid fermentation, polysaccharide, structural characteristic, prebiotic effects

## Abstract

In the present study, medium composition of *Cordyceps farinosa* Cf-GZU06 mycelial fermentation was optimized using response surface methodology. Both intracellular polysaccharides (IPS) and extracellular polysaccharides (EPS) from *C. farinosa* Cf-GZU06 were obtained and investigated for their structural properties and prebiotic potential. The final medium consisted of 76.170 g/L glucose, 11.056 g/L peptone, 22.201 g/L yeast extract, 0.600 g/L MgSO_4_·7H_2_O and 1.400 g/L KH_2_PO_4_ with the maximum mycelial biomass reaching 44.797 g/L. Structural characteristics showed that EPS and IPS had similar primary structures but different fine structures, especially the aggregate states and microstructures. The primary structures of these two polysaccharides showed limited susceptibility in in vitro digestion with no major changes in their molecular weight (MW) profiles, while the fine structure could be altered during digestion process with less than 17% (*w*/*w*) reducing sugar detected. Both EPS and IPS could reshape the fecal microbial composition toward a susceptible balance. The production of short-chain fatty acids (SCFAs) including acetate, propionic and butyric acid was significantly increased, and the total SCFAs was increased to 315.18 mM and 302.81 mM by EPS and IPS respectively compared to inulin (279.06 mM). Microbial metabolic profiles showed that EPS and IPS had totally different impacts on bacterial metabolism. These results suggested that EPS and IPS exhibited a potential prebiotic effect under in vitro conditions, supporting their further evaluation as prebiotic candidates.

## 1. Introduction

*Cordyceps* primarily consists of ascomycetes parasitic fungi which propagate through parasitism mainly on insects, arthropods and a few on other fungi [1,2]. Among the genus, *Cordyceps sinensis* (*Ophiocordyceps sinensis*) and *Cordyceps militaris*, known as “Dong Chong Xia Cao” and “Yong Chong Cao” respectively in Chinese, are the most two well-known and widely utilized species for their multiple pharmacological properties such as antioxidant effects, antitumor and immunomodulation activities [3]. *C. sinensis* has been used as traditional Chinese medicine for centuries, and its wild scarcity has driven the development of *C. militaris* as a sustainable alternative with a global market of USD 1.36 billion in 2025 [4]. *Cordyceps farinosa* (the original name *Isaria farinosa* and the new name *Ophiocordyceps farinosa*) is often applied as a biocontrol agent due to its wide host range and high mammal safety [5]. *C. farinosa* can efficiently infect *Thitarodes armoricanus* larvae competitively with *C. sinensis* [6]. Recent studies have found several similar bioactive metabolites generated by *C. farinosa* to that by *C. sinensis* and *C. militaris,* including triterpenoids and flavonoids, which indicate that *C. farinosa* might also have competitive functional properties with *C. sinensis* and *C. militaris* [7,8]. However, few studies have investigated the bioactive functions of *C. farinosa*.

Polysaccharides are acknowledged as the principle bioactive ingredients of *Cordyceps* with numerous pharmacological functions including immunomodulatory, hypolpidemic and antitumor effects [9,10]. *Cordyceps* sp. produce two types of polysaccharides: extracellular polysaccharides, which are secreted by fungal cells into the surrounding medium, and intracellular polysaccharides, which accumulate within the cytoplasm. These extracellular and intracellular polysaccharides usually have relatively high molecular weight (MW) with MW ranging from 10 kDa to 10^4^ kDa and consist of similar monosaccharides, mainly mannose, glucose and galactose [10]. Mycelium fermentation has been reported recently to show high efficiency in obtaining both intracellular and extracellular polysaccharides from various *Cordyceps* sp. For example, the intracellular polysaccharide CS-Pp with a yield of 4.2% (*w*/*w*) was isolated from the fungi Cs-4 [11], the intracellular polysaccharide HSWP-2a with a yield of 4.38% (*w*/*w*) was extracted from Cs-C-Q80 [12], and the extracellular polysaccharide EPS-LM-1 with a yield of 0.074 g/L was obtained from Cs-HK1 [13], which were comparable to the polysaccharides extracted from plants with yields of 0.75–9.71% (*w*/*w*) [14,15,16]. In contrast to these well-studied polysaccharides from other *Cordyceps* sp., the corresponding polysaccharides from *C. farinosa* have not yet been identified so far.

The prevalence of intestinal dysbiosis is rising globally, contributing to a range of chronic diseases including obesity, inflammatory bowel disease and metabolic syndrome [17,18]. The market of prebiotics, which is applied as an effective and safe intervention that restores gut environmental balance, has experienced substantial growth at approximately USD 11.31 billion in 2025 and is projected to grow at a compound annual growth rate (CAGR) of 14.55% from 2025 to 2034 [19]. Natural polysaccharides from different sources like fungus with high resistance in the human digestive tract are considered an important source of potential prebiotics [20]. Recent investigation of *C. sinensis* showed that the polysaccharides could modulate gut microbial composition and regulate bacterial metabolism [21,22]. Structural characteristics of polysaccharides such as monosaccharide composition, linkage types, and the size or molecular weight are the essential factors affecting their interaction with gut microbiota. However, limited reports have focused on the structure–activity relationships between *Cordyceps* polysaccharides and their prebiotic functions, and nearly none of the studies have illustrated the prebiotic potential of the polysaccharides from *C. farinosa*.

In this study, the medium composition of *C. farinosa* in liquid fermentation was first optimized based on mycelial biomass as the indicator by response surface methodology. Both IPS and EPS were then obtained for their structural characterization. Digestibility of the IPS and EPS were evaluated depending on the changes in reducing sugar content and molecular weight during in vitro digestion. Fecal fermentation was conducted to investigate the modulation effects of the polysaccharides on gut microbiota composition. The analysis on short-chain fatty acids and metabolic profiles was performed to explore their influence on bacterial metabolism.

## 2. Materials and Methods

### 2.1. Materials and Chemicals

*C. farinosa* Cf-GZU06 fresh organism was collected on Mopan Mountain in Xinping County, Yunnan Province, southwest China (23°97′ N, 102°03′ E) and the mycelium was isolated by Prof. Ting-Chi Wen from Guizhou University (Guiyang, China). *C. farinosa* Cf-GZU06 mycelium as a gift from Prof. Ting-Chi Wen for our lab was identified both morphologically (Figure S1) and genetically by Sangon Biotech (Shanghai) Co., Ltd. (Shanghai, China). Dextran molecular weight standards ranging from 1 kDa to 670 kDa, monosaccharide standards (arabinose, mannose, fucose, glucose, galactose, glucuronic acid, galacturonic acid, rhamnose, ribose, and xylose) (Sigma-Aldrich, St. Louis, MO, USA) and short-chain fatty acid standards (acetic acid, propionic acid, *n*-butyric acid, *i*-butyric acid, *n*-valeric acid, *i*-valeric acid) (Shanghai Aladdin Biochemical Technology Co., Ltd., Shanghai, China) were sourced commercially. All the chemicals and reagents in this study were analytical or high-performance liquid chromatography (HPLC) grade.

### 2.2. Culture Medium Optimization

Liquid culture was carried out in shake-flasks according to a previous report with certain modifications [23]. *C. farinosa* Cf-GZU06 was first incubated on potato dextrose agar (PDA) at 20 °C for 7 d. A visible quantity of the mycelium was picked up using an inoculation loop and then transferred into a 250 mL Erlenmeyer flask containing 50 mL basal medium (40 g/L glucose, 5 g/L peptone, 1 g/L KH_2_PO_4_, 0.5 g/L MgSO_4_·7H_2_O, 10 g/L yeast extract). After culture at 150 rpm for 7 d, the starter culture (5%, *v*/*v*) in basal medium was inoculated into the fermentation medium at 150 rpm for response surface methodology (RSM) analysis. The fermentation broth was centrifuged at 8000 rpm (4472× *g*) for 20 min and the fungal mycelia were collected, washed, centrifuged (4472× *g*, 20 min), freeze-dried and used as the indicator for RSM analysis. RSM analysis including Plackett–Burman design (PBD), path of steepest ascent and Box–Behnken design (BBD) were conducted on the software Design-expert version 13.

#### 2.2.1. Plackett–Burman Design (PBD)

Six variables including culture time (A), glucose (B), peptone (C), yeast extract (D), MgSO_4_·7H_2_O (E) and KH_2_PO_4_ (F) were applied for the linear model to evaluate the critical factors affecting fungal biomass. Two different levels (low and high level coded as −1 and +1, respectively) of each factor were set as shown in Table S1. The low (6 d) and high level (10 d) of culture time were chosen to cover the potential effects of both short and long culture time on mycelial growth. The low and high level of the five other factors was based on their concentration in basal medium as medium level. In total, twelve experimental runs with each in triplicate were conducted (Table S2).

#### 2.2.2. Path of Steepest Ascent

According to the results of PBD (Table S3), three factors (glucose, peptone and yeast extract) mostly affecting the growth of *C. farinosa* mycelium were chosen for path of steepest ascent. Step direction and step size of the climbing were dependent on the biomass increasing direction and the levels in PBD, respectively. Nine experimental runs with each in triplicate were designed (Table S4).

#### 2.2.3. Box–Behnken Design (BBD)

Three levels (low, medium and high level coded as −1, 0 and +1, respectively) of the three factors (glucose, peptone and yeast extract) selected by PBD were set to further approach the optimal medium composition (Table S5). The concentration of the three factors yielding maximum fungal biomass in the path of steepest ascent were applied as medium level [24]. A total of seventeen experimental runs with each in triplicate were conducted (Table S6).

### 2.3. Extraction and Purification of Polysaccharide

Polysaccharide extraction was based on previous reports with certain changes [16,23]. Mycelial fermentation broth was centrifuged at 8000 rpm (4472× *g*) for 20 min. Both supernatant and mycelium were collected for extracellular (EPS) and intracellular polysaccharide (IPS) extraction, respectively. For EPS extraction, 5 volumes of anhydrous ethanol were added into the supernatant and maintained at 4 °C overnight for complete precipitation. The mixture was centrifuged at 8000 rpm (4472× *g*) for 10 min. The precipitate was redissolved in distilled water, decolorized using AB-8 macro-porous resin, added with 1.5% (*w*/*w*) papain and incubated at 55 °C for 3 h. The reactant was deproteinized using Sevag method. Rotary evaporation and dialysis (3.5 kDa) were applied to exclude organic solvents and small molecules. The products were then freeze-dried to obtain EPS. For IPS extraction, the mycelium was freeze-dried, pulverized and screened with a 100-mesh sieve to obtain *C. farinosa* mycelial powder. The dried powder was mixed with distilled water at a ratio of 1:20 (*w*/*v*). The mixture was boiled for 4 h, followed by centrifugation at 8000 rpm (4472× *g*) for 15 min. The supernatant was collected, mixed with 5 volumes of anhydrous ethanol and maintained at 4 °C overnight. The precipitate was separated by centrifugation (8000 rpm, 4472× *g*, 10 min), redissolved in distilled water, decolorized and deproteinized using the same procedures as EPS to attain IPS.

### 2.4. Physicochemical and Structural Properties of Polysaccharide

#### 2.4.1. Physical Properties

Anthrone test was applied to determine the total carbohydrate content of EPS and IPS with glucose as a standard [13]. Bradford method was used for the protein content measurement using bovine serum albumin (BSA) as a standard. The moisture content of polysaccharide samples (0.5 g) was analyzed by a moisture analyzer (Mettler Toledo, HR83-P, Greifensee, Switzerland) at 20 °C. The average particle size and Zeta potential of EPS and IPS aggregation in the solution (1 mg/mL) was measured on a Zetasizer Nano-ZS (Malvern Instruments Ltd., Worcestershire, UK) at 20 °C.

#### 2.4.2. Molecular Weight Distribution

Molecular weight (MW) distribution of EPS and IPS was determined by high-performance gel permeation chromatography (HPGPC) according to a previous report with minor changes [14]. The analysis was conducted on a Waters 1515 isocratic pump system connected to a 2414 refractive index detector. Two tandem columns, Ultrahydrogel 250 (300 mm × 7.8 mm, 6 μm) and 2000 (300 mm × 7.8 mm, 12 μm) (Waters Co., Milford, MA, USA), were applied at 40 °C for separation. Sodium chloride (0.2 M) containing 0.02% (*w*/*w*) sodium azide at 0.6 mL/min was used as the mobile phase. Polysaccharide sample (3 mg) was dissolved in 1 mL mobile phase and filtered through a 0.45 μm membrane before injection. Dextran MW standards (1, 5, 12, 25, 50, 80, 270, 410 and 670 kDa) were applied for MW calibration.

#### 2.4.3. Monosaccharide Composition

Monosaccharide composition of samples was determined using 1-phenyl-3-methyl-5-pyrazolone high-performance liquid chromatograph (PMP-HPLC) method as described previously with certain modifications [25]. Polysaccharide samples (3 mg) were completely hydrolyzed to monosaccharide in 2 mL 2M trifluoroacetic acid (TFA) at 110 °C for 6 h. The hydrolysate was washed thrice by methanol (200 μL), dried by nitrogen-blowing and redissolved in 1 mL distilled water. The hydrolysate solution (450 μL) was mixed with NaOH (0.3 M) at an equal volume and reacted with 450 μL 0.5 M PMP at 70 °C for 100 min. HCl (0.3 M, 450 μL) was added to stop the reaction. The aqueous layer was collected, washed by chloroform three times and filtered through a 0.45 μm membrane for chromatography analysis. HPLC was conducted with a XBridgeTM C18 column (150 mm × 4.6 mm, 5 μm) on an Agilent 1260 Infinity II system (Agilent Technologies, Waldbronn, Germany) at 25 °C. The mobile phase consisted of potassium phosphate-buffered saline (0.05 M, pH 6.8) containing 15% (*v*/*v*) (solution A) and 40% (*v*/*v*) acetonitrile (solution B).

#### 2.4.4. FT-IR, UV Spectroscopy and Morphology Analysis

The infrared spectra of EPS and IPS were recorded on a Frontier^TM^ Fourier transform infrared (FT-IR) spectrometer (PerkinElmer, Waltham, MA, USA). Samples (2 mg) were mixed with KBr, ground and pressed. The data were collected in the 4000–400 cm^−1^ wavelength range. Ultraviolet–visible (UV) spectra of sample solution (0.5 mg/mL) were recorded on a UV-1900i spectrophotometer (Shimadzu, Kyoto, Japan) in the wavelength range of 200–600 cm^−1^.

The microstructure of EPS and IPS was monitored by scanning electron microscopy (SEM) on a Sigma 360 system (ZEISS, Oberkochen, Germany). Samples (10 mg) were evenly dispersed on a conductive adhesive and coated with gold by a Quorum SC7620 sputter (Quorum Technologies Ltd., Ashford, Kent, UK).

### 2.5. In Vitro Digestibility

#### 2.5.1. Simulated Digestion Fluid Preparation

The simulated saliva (SSF), gastric (SGF) and intestinal digestion fluids (SIF) were prepared by mixing electrolyte stock solution with various enzymes according to a well-documented protocol [26]. A series of salt solutions [0.5 M KH_2_PO_4_, 0.5 M KCl, 0.5 M (NH_4_)_2_CO_3_, 0.15 M MgCl_2_(H_2_O)_6_, 1 M NaHCO_3_, 2 M NaCl] for electrolyte stock solution of SSF, SGF and SIF were prepared and mixed according to Table S7. HCl (pH 6) and NaOH (pH 1) were used for pH adjustment. SSF was obtained by mixing SSF electrolyte stock solution (4 mL) with CaCl_2_ (37.8 g/mL, 0.025 mL), α-amylase (500 U/mL, 0.75 mL) and distilled water (0.225 mL), SGF was attained by mixing SGF electrolyte stock solution (3.2 mL) with CaCl_2_ (37.8 g/mL, 0.002 mL), pepsin (75,000 U/mL, 0.214 mL) and distilled water (0.548 mL), and SIF was prepared by mixing SIF electrolyte stock solution (3.2 mL) with CaCl_2_ (37.8 g/mL, 0.008 mL), trypsin (5000 U/mL, 0.16 mL), bile salt (4 mg/mL, 0.2 mL) and distilled water (0.432 mL). All the simulated digestion fluids were freshly prepared just before use.

#### 2.5.2. Digestibility Analysis

The digestibility of EPS and IPS was analyzed according to Tu et al. with certain changes [15]. Polysaccharide solution (8 mg/mL) was mixed with SSF at an equal volume. The mixture was pH adjusted to 7 and incubated at 37 °C for 5 min. The oral digestate was pH adjusted to 3, mixed with SGF at a ratio of 1:1 (*v*/*v*) and maintained at 37 °C for 120 min. The gastric digestate was pH adjusted to 7 and mixed with SIF at an equal volume. After reaction at 37 °C for 120 min, the mixture was boiled for 10 min to inactivate enzymes. HCl (pH 6) and NaOH (pH 1) were used for pH adjustment. The digestates at oral digestion 0, 5 min, gastric digestion 60, 120 min and intestinal digestion 60, 120 min were collected and diluted to 1 mg/mL (polysaccharide concentration) by H_2_O for reducing sugar content and molecular weight analysis. Reducing sugar content was determined using 3,5-dinitrosalicylic acid (DNS) method [27]. Molecular weight changes in the digestates were analyzed using HPGPC as described in Section 2.4.2.

### 2.6. Fecal Fermentation

The in vitro culture of fecal microbiota was conducted according to our previous report [16]. Fecal samples were collected from three healthy adult donors with age of 20–30 years (1 male and 2 females), who had not taken antibiotics, prebiotics and probiotics, and no gastrointestinal diseases for the past three months. Fresh fecal materials were collected and immediately stored under an anaerobic condition. The fecal samples from the three donors were mixed at a ratio of 1:1:1 (*w*/*w*). After diluting with phosphate-buffered saline (PBS, pH 7.4) at 1:9 ratio (*w*/*w*), the mixture was homogenized for 5 min and then centrifuged (500 rpm, 4472× *g*, 10 min) to remove large particles. The supernatant remained as fecal inoculum. Anaerobic condition was generated by anaerobic jars and anaerobic gas-generating sachets (AnaeroGen^TM^, Oxoid Ltd., Basingstoke, Hampshire, UK).

Basal medium (1 L) for in vitro fermentation consisted of 2 g yeast extract, 2 g peptone, 0.1 g NaCl, 2 g NaHCO_3_, 0.04 g KH_2_PO_4_, 0.04 g K_2_HPO_4_, 0.01 g CaCl_2,_ 0.01 g MgSO_4_·7H_2_O, 0.5 g bile salt, 0.5 g L-cysteine, 10 mg vitamin K1, 0.01 g hemin, 2 mL Tween-80 and 1 mg resazurin. Polysaccharide samples at 5 mg/mL were dissolved in the basal medium, stirred overnight, pH adjusted to 6.8 and autoclaved at 121 °C for 20 min to prepare culture medium. The culture medium (5.4 mL) which was kept in anaerobic condition at least 2 h before use and fecal inoculum (0.6 mL) was mixed in a 10 mL tube and incubated at 37 °C, 150 rpm for 48 h in anaerobic condition. The basal medium without any additional carbon sources was used as blank and inulin was applied as a prebiotic control.

### 2.7. Microbial Composition Analysis

The fermented culture was centrifuged at 12,000 rpm (13,523× *g*) for 10 min and the sediments were collected for microbial composition analysis. Bacterial DNA was extracted using an E.Z.N.A.^®^ soil DNA kit (Omega Bio-tek, Norcross, GA, USA) following the manufacturer’s instructions. DNA concentration was measured on a NanoDrop 2000 spectrophotometer (Thermo Fisher, Waltham, MA, USA). 16S rRNA analysis was performed by Shanghai Majorbio Bio-pharm Technology Co., Ltd. (Shanghai, China). Bacterial taxonomy was assigned using the Silva (Release 138) and RDP (Release 11.5) databases.

### 2.8. Short-Chain Fatty Acid (SCFA) Analysis

SCFAs in the culture supernatant (centrifuged at 12,000 rpm, 13,781× *g*, 10 min) were detected by gas chromatography (GC) [23]. The supernatant (1.2 mL) was acidified with 240 mL 50% (*v*/*v*) H_2_SO_4_ at 4 °C for 1 h. The mixture was added with 1.2 mL ethyl acetate and vortexed for 10 min. After centrifugation at 8000 rpm (6123× *g*) for 5 min, the organic layer was collected and filtered through a 0.22 μm membrane before injection into the GC system. Diethylacetic acid (120 mL, 7.9 mM) was used as an internal standard. Analysis was performed on an Agilent 7980B GC system connected to a flame ionization detector (FID) (Agilent Technologies Inc., Santa Clara, CA, USA). SCFAs were separated on a fused silica capillary column (DB-FFAP 123-3232, 30 mm × 0.25 mm, Agilent Technologies Inc.). Ultrapure nitrogen gas applied as the mobile phase flowed at 0.6 mL/min. The initial oven temperature was set at 105 °C for 3 min, then raised to 170 °C gradually by 10 °C/min, and finally raised to 240 °C at the rate of 70 °C/min. Six SCFA standards including acetic acid, propionic acid, i-butyric acid, n-butyric acid, i-valeric acid and n-valeric acid were used for identification and quantification.

### 2.9. Metabolomic Analysis

Microbial metabolites in the culture supernatant (centrifuged at 12,000 rpm, 13,523× *g*, 10 min) were analyzed using liquid chromatography–mass spectrometry (LC-MS) by Shanghai Majorbio Bio-pharm Technology Co., Ltd. (Shanghai, China) according to a well-documented report [28]. Briefly, the supernatant (200 μL) was mixed with 800 μL extraction solvent (acetonitrile: methanol = 1:1, *v*/*v*) containing an internal standard L-2-chlorophenylalanine at 0.02 mg/mL. The mixture was vortexed, ultrasonic treated (40 kHz, 30 min, 5 °C) and maintained at −20 °C for 30 min. After centrifugation (13,000× *g*, 15 min), the supernatant was nitrogen-dried and redissolved in 50% acetonitrile. The extraction procedures were repeated twice before analysis. Metabolic profile analysis was conducted on an Agilent UHPLC-Q Exactive HF X system connected to an ACQUITY UPLC HSS T3 column (100 mm × 2.1 mm, 1.8 µm; Waters, Milford, MA, USA). Solution A containing 5% (*v*/*v*) acetonitrile and 0.1% (*v*/*v*) formic acid and solution B containing 47.5% (*v*/*v*) acetonitrile, 47.5% (*v*/*v*) isopropanol and 0.1% (*v*/*v*) formic acid were applied as mobile phase.

The original data was processed using the software Progenesis QI v3.0 (WatersCorporation, Milford, USA). The human metabolome database (HMDB, http://www.hmdb.ca/, accessed on 10 November 2025) and Metlin database (https://metlin.scripps.edu/, accessed on 10 November 2025) were applied to identify the metabolites. The differential metabolic compounds between each two groups (VIP > 1.0, *p*-value < 0.05 and foldchange value > 1 or <1) were then analyzed based on KEGG database (http://www.genome.jp/kegg/, accessed on 10 November 2025).

### 2.10. Statistical Analysis

All experiments in this study were carried out three times, and most data were expressed as mean ± standard deviation (SD). Statistical evaluations, including ANOVA, *t*-test, and OPLS-DA, were conducted using SPSS (version 24.0) and the free online Majorbio Cloud Platform (cloud.majorbio.com). A *p*-value of less than 0.05 was considered statistically significant.

## 3. Results and Discussion

### 3.1. Optimization of Liquid Medium Compositions

According to the fungal biomass obtained from the 12 runs in the PBD test (Table S2), the first-order equation was attained:

Y = 1.1800 − 0.0190A + 0.01763B + 0.0144C + 0.0604D + 0.0029E + 0.0114F(1)

An analysis of variance (ANOVA) was performed on the six factors present in the equation (Table S3). The *p*-value of the model was less than 0.0001, indicating that the model with mycelial biomass as the response was statistically significant. The *p*-values of culture time (A), glucose (B), peptone (C) and yeast extract (D) were all less than 0.01, suggesting their significant influence on biomass production. In this study, only medium composition was focused; therefore, glucose, peptone and yeast extract were chosen as critical factors. Both MgSO_4_·7H_2_O (E) and KH_2_PO_4_ (F) had a positive effect on biomass, thus they were set at their maximum values in the PBD test (0.6 g/L and 1.4 g/L, respectively) for the further analysis, and culture time with negative effect was set at its minimum value (6 d).

Based on the path of steepest ascent (Table S4), mycelial biomass approached the maximum value (38.680 g/L) with the concentration of glucose, peptone and yeast extract at 75 g/L, 11 g/L and 22 g/L, respectively, which were applied as medium level in BBD test (Table S5). According to the biomass yields in the 17 runs of BBD test (Table S6), the second-order equation was obtained:

Y = 2.2300 + 0.0437B + 0.0206C + 0.0381D − 0.0473BC − 0.0328BD + 0.0050CD − 0.1614B^2^ − 0.1391C^2^ − 0.1726D^2^ (2)

As shown in Figure S2a, the points in the normal plot of residuals generally followed the reference diagonal line with only slight deviations at both ends, indicating that the residuals of this model approximately followed a normal distribution. Figure S2b shows a scatter plot of residuals versus predicted values. The residuals were randomly scattered around the zero line without any funnel-shaped pattern, suggesting that the error variance was constant, and the model exhibited good homoscedasticity. The plot of residuals versus run order (Figure S2c) showed no obvious trend, drift, or serial structure, indicating good independence of the model residuals. The predicted values also showed good agreement with the actual observed values (Figure S2d). The adequate precision of the model was 16.6690, which was far above the generally recommended minimum value of 4, indicating that the model had sufficient signal-to-noise ratio and discriminative ability. The coefficient of variation (CV%) was 1.75, suggesting good experimental repeatability and high model stability. The model with *p*-value less than 0.0001 and lack of fit with *p*-value of 0.9330 (>0.05) (Table S8) indicated the statistical significance of the model and the high correlation between the model and biomass, respectively. Both the coefficient of determination R^2^ (0.9787) and the adjusted R^2^ (0.9514) of the model were close to 1, again demonstrating a high correlation between the experimental and predicted values. The difference between the predicted R^2^ (0.9382) and adjusted R^2^ was only 0.0132, suggesting that the model not only exhibited good fitting ability but also possessed a strong predictive capability.

**Figure 1:**
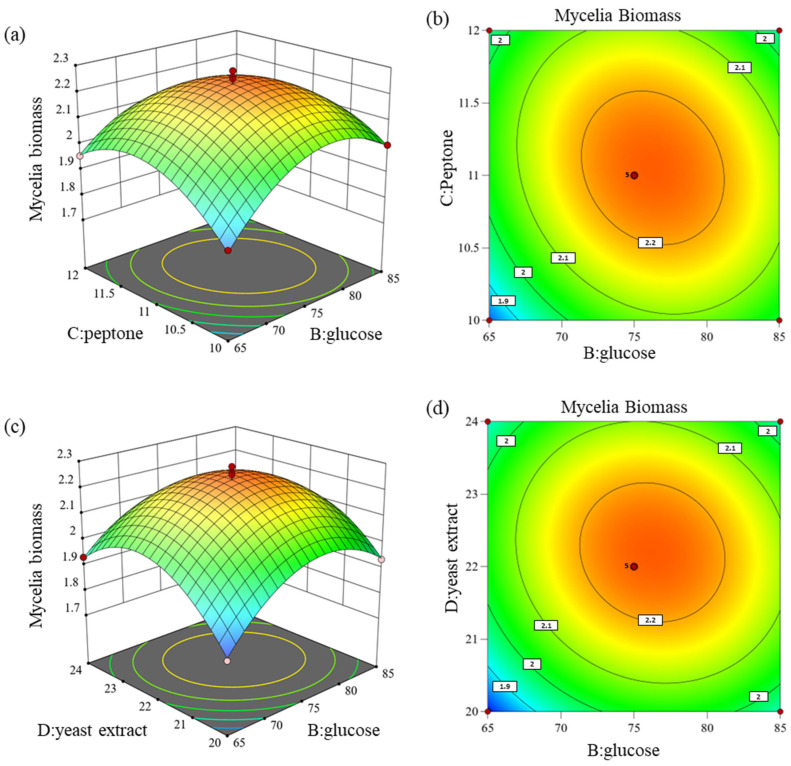

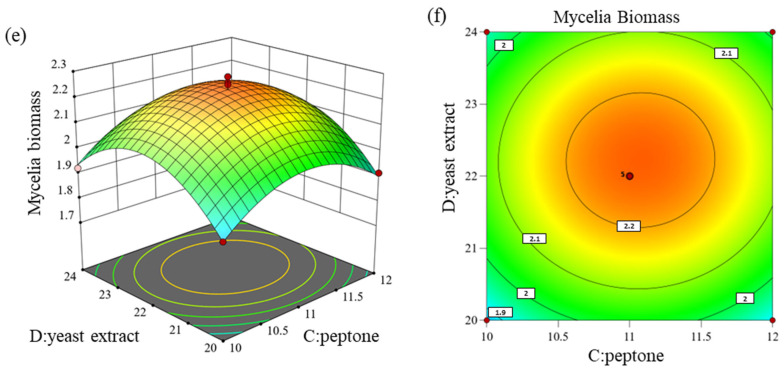
Response surface plots of three critical factors on *C. farinosa* Cf-GZU06 mycelia biomass. Three-dimension response surface plot of (**a**) glucose and peptone, (**c**) glucose and yeast extract, (**e**) peptone and yeast extract. Two-dimension contour plot of (**b**) glucose and peptone, (**d**) glucose and yeast extract, and (**f**) peptone and yeast extract.

The interactions among glucose, peptone and yeast extract illustrated that glucose had a more significant influence on biomass than the other two factors (Figure 1). Based on the BBD model, the mycelial biomass would reach a maximum value of 44.797 g/L with the concentrations of glucose, peptone, and yeast extract at 76.170 g/L, 11.056 g/L, and 22.201 g/L, respectively, which was 1.9 times the biomass obtained from the basal medium before optimization. The optimized medium was applied in the mycelial fermentation to actually obtain 44.270 g/L of the biomass with a relative error of 1.19% compared to the predicted value. Based on the initial glucose concentration, the biomass yield was calculated to be 58.12% (gram mycelium per gram of glucose, 44.270 g/L ÷ 76.170 g/L). The carbon (glucose) to nitrogen (peptone and yeast extract) (C/N) ratio (2.3:1) in this study was much lower compared to the liquid fermentation of other *Cordyceps* sp., such as *C. sinensis* Cs-HK1 (2.7:1) and *C. militaris* C738 (6:1) [29,30]. In terms of biomass yield based on initial glucose concentration, *C. sinensis* Cs-HK1 produced 20.9 g/L biomass with 40 g/L glucose giving a yield of 52.25%, while *C. militaris* C738 produced 12.7 g/L biomass with 60 g/L glucose, giving a yield of 21.17%. The yield of *C. farinosa* Cf-GZU06 was higher than both. Usually, a high concentration of nitrogen sources might inhibit fungal growth. However, even with a low C/N ratio, *C. farinosa* Cf-GZU06 achieved a higher biomass yield than *C. sinensis* Cs-HK1 and *C. militaris* C738. These differences might result from strain-specific growth characteristics, as different *Cordyceps* sp. exhibit substantial variation in growth kinetics. The high yield suggested that *C. farinosa* Cf-GZU06 would be a potential alternative of *C. sinensis* and *C. militaris* for industrial production.

### 3.2. Chemical Composition and Physical Properties of EPS and IPS

**Figure 2:**
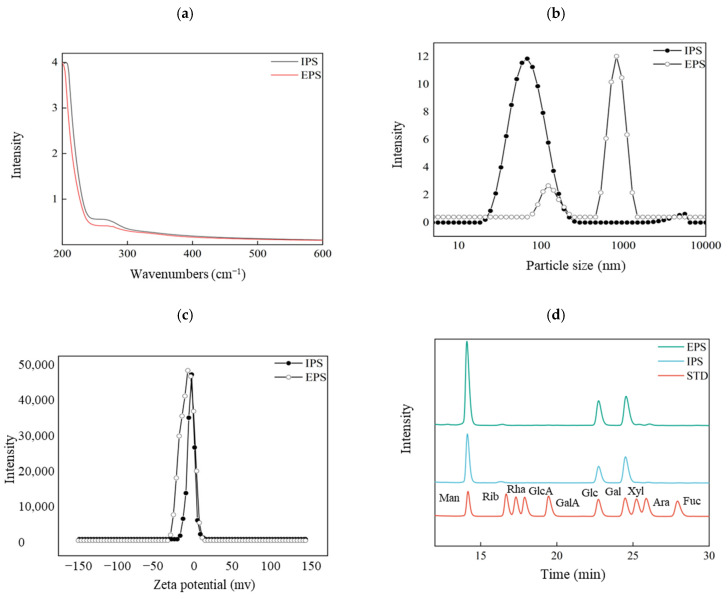

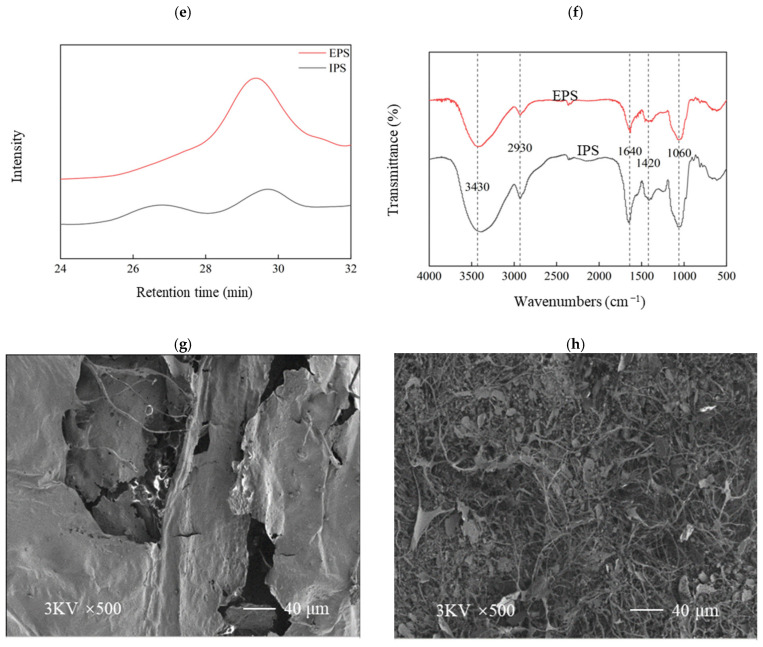
Physicochemical properties of IPS and EPS. (**a**) UV spectra, (**b**) particle size distribution, (**c**) Zeta potential, (**d**) HPLC profiles, (**e**) HPGPC profiles, (**f**) FT-IR spectra and SEM image of (**g**) IPS and (**h**) EPS with magnification factors at 500×. STD, standard; Ara, arabinose; Fuc, fucose; Gal, galactose; GalA, galacturonic acid; Glc, glucose; GlcA, glucuronic acid; Man, mannose; Rib, ribose; Rha, rhamnose; Xyl, xylose.

Table 1 shows the yield and major chemical content of the polysaccharides. The yield of EPS and IPS was 0.60 g/L and 1.63% (*w*/*w*), respectively. The different units used for the yield were based on the different preparation process of the polysaccharides. The carbohydrate content (*w*/*w*) of EPS (75.86%) and IPS (61.94%) fell within the range reported for other *Cordyceps*-derived polysaccharides, such as *C. sinensis* (70%) and *C. militaris* (66%) [23,31], suggesting that both EPS and IPS were carbohydrate-rich macromolecules. The moisture content was 9.24% for EPS and 8.40% for IPS. The residual proteins were low in EPS (1.88%) and IPS (4.60%), which could be confirmed by UV spectra (Figure 2a), indicating acceptable purity for further functional evaluation. EPS had two forms in the solution with particle size of 846.75 nm and 128.60 nm (Table 1 and Figure 2b). Polysaccharides, as macromolecules, were highly prone to forming aggregates in aqueous solutions [32]. The larger one with 846.75 nm was most likely a secondary aggregate generated by the clustering of the smaller ones. The average particle size of IPS was 61.19 nm. This indicated that EPS and IPS had different aggregate states in aqueous solution. These were comparable to the polysaccharides from different natural sources with particle size ranging from approximately 10 nm to 1000 nm [33,34]; Zeta potential of EPS and IPS was −8.27 mV and −2.71 mV (Table 1 and Figure 2c), respectively, suggesting the negative charges of the surface of the polysaccharide aggregation in aqueous solution. The absolute values of Zeta potential were low (<±15 mV), indicating that EPS and IPS had limited colloidal stability and they might undergo further aggregation or sedimentation over time. This inherent instability presented a challenge for incorporating these polysaccharides into liquid food products (e.g., beverages or functional drinks), where sedimentation or phase separation would negatively impact product quality and consumer acceptance. Consequently, formulation strategies to enhance their dispersion stability would be necessary for such applications. Compared to EPS (−8.27 mV), IPS (−2.71 mV) had relatively low negative charges. This suggested IPS might experience weaker electrostatic repulsion with bacterial membranes, potentially favoring its initial adhesion and uptake by gut microbes.

### 3.3. Monosaccharide Composition and Molecular Weight Distribution of EPS and IPS

As shown in Figure 2d, both EPS and IPS consisted of glucose, galactose and mannose. The ratio of glucose:galactose:mannose for EPS and IPS was 1:1.257:2.633 and 1:1.706:2.167, respectively, which was similar to polysaccharides from various *Cordyceps* spp. [35,36,37]. Figure 2e and Table 2 show the molecular weight (MW) distribution of EPS and IPS. EPS had a broad peak ranging from 25.267 min to 31.862 min with an average MW of 25.46 kDa. There might be several fractions existing in EPS that overlap to exhibit the broad peak. There were two peaks in IPS with MW of 136.82 kDa and 22.96 kDa, respectively. This indicated that IPS contained higher proportion of high MW fractions than EPS.

### 3.4. FT-IR Analysis

Figure 2f illustrates the FT-IR spectra of the polysaccharides. The infrared absorption profiles of EPS and IPS were nearly identical with only intensity differences observed, suggesting that EPS and IPS had similar functional groups in their primary structures. The broad and intense peak at 3430 cm^−1^ was the characteristic absorption of polysaccharides, representing the O-H stretching vibration from intermolecular or intramolecular hydrogen bonds [38]. The peak at 2930 cm^−1^ was attributed to the C-H stretching vibration [39]. EPS and IPS were neutral polysaccharides without any uronic acid content and they both contained a certain amount of moisture; therefore, the peak at 1640 cm^−1^ highly corresponded to the O-H bending vibration of water molecules. The absorption peak at 1060 cm^−1^ was related to the bending vibrations of C-O-C in pyranose rings [40]. These results together with the monosaccharide composition and molecular weight distribution suggested that EPS and IPS had similar primary structures.

### 3.5. Microstructure of EPS and IPS

Figure 2g and Figure 2h present the morphology of IPS and EPS, respectively. IPS showed a sheet-like structure with a relatively smooth and flat surface, and there were certain wrinkles and protrusions on the surface, while EPS showed an irregular and disordered network structure with some fragmented sheets inserted in the network. The porous surface suggested that EPS had stronger capability of water absorption, which was consistent with the result that EPS contained higher moisture content than IPS (Table 1). These results indicated that polysaccharides from different locations (intra- or extracellular) might have completely different fine structures, even if they were derived from the same fungal source with similar primary structures.

### 3.6. Digestibility of EPS and IPS

Soluble starch which would be digested in the human digestive tract was used as a positive control (Figure S3 and Table S9). The reducing sugar content in soluble starch increased from 9.03% to 33.85%. Although the retention time of the major peak of soluble starch in HPGPC profiles shifted only from 34.825 min to 35.289 min, the peak area had a notable decrease after digestion. At 0 min of SSF digestion (Figure S3, light green line), soluble starch had a broad unsymmetric weak peak around 32 min. After 5 min digestion in SSF (Figure S3, red line), this peak disappeared and the major peak at 34.825 min showed an increase in peak area. This indicated that some high MW fractions in soluble starch degraded to show a relatively lower MW. The peak shifted to 34.901 min with a sharp decrease in peak area after SGF digestion, indicating an extensive hydrolysis. The produced oligosaccharides and monosaccharides most likely eluted near the solvent peak or beyond the total volume of the column, and thus no new peaks appeared in the MW profiles. As in vitro digestion proceeded, the peak continued to shift to 35.289 min after SIF digestion. These results suggested the degradation of soluble starch during the whole in vitro digestion.

**Figure 3:**
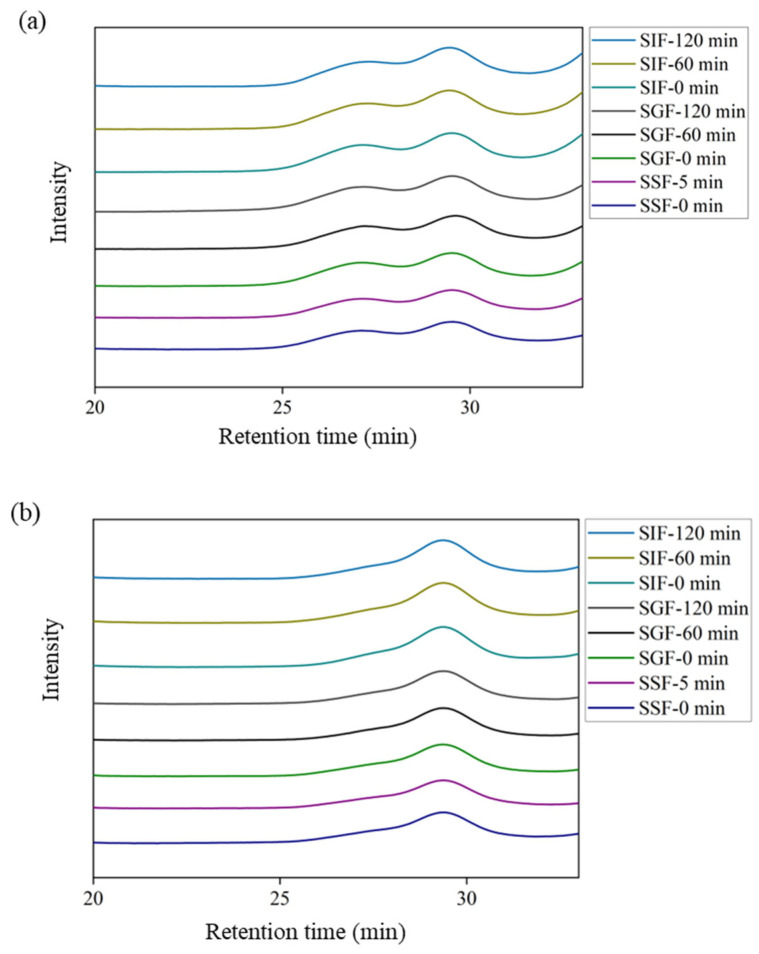
Molecular weight distribution of (**a**) IPS and (**b**) EPS during in vitro digestion. SSF, simulated saliva digestion fluid; SGF, simulated gastric digestion fluid; SIF, simulated intestinal digestion fluid.

Table 3 and Figure 3 show the reducing sugar content and molecular weight changes in the polysaccharides during in vitro digestion, respectively. EPS and IPS contained 2.47% and 2.60% (*w*/*w*) reducing sugar, respectively (Table 3). There were no significant changes in reducing sugar content and MW profiles of these two polysaccharides in SSF (Table 3 and Figure 3), suggesting high resistance of EPS and IPS to the oral digestion. The reducing sugar content increased to 5.56% and 5.66% in EPS and IPS, respectively, at 0 min of SGF digestion. The DNS method was applied in this study to determine reducing sugar content, which was susceptible to pH [41]. The digestion reaction had not yet started at 0 min; therefore, the increase was likely due to the changes in buffer solution and pH, rather than the degradation of polysaccharides. This result could be confirmed both by the reducing sugar content in the control (polysaccharide after sequential mixing with SGF and SIF buffer but without any enzymes), which was 5.34% and 5.48% for EPS and IPS respectively in SGF buffer and 10.97% and 11.02% for EPS and IPS respectively in SIF buffer, and by the MW profiles that had no substantial shift in their peaks. Besides the retention, the peak area of EPS and IPS also remained nearly constant without notable changes. After digestion in SGF, the reducing sugar content had a slight decrease (4.96% in EPS and IPS), which probably resulted from the dehydration of reducing sugar in acidic environment. This was similar to the polysaccharides derived from *Cordyceps sinensis* Cs-HK1 with less than 5% (*w*/*w*) reducing sugar after gastric digestion [33]. During SIF digestion, the reducing sugar content of EPS increased from 11.16% to 12.30% in the first 60 min and further to 13.86% in the subsequent 60 min. IPS showed a similar trend, increasing from 11.94% to 14.42% in the first 60 min and then to 16.10% in the next 60 min. However, the HPGPC profiles revealed no apparent shifts in MW distribution for either polysaccharide, and the peak areas remained constant throughout SIF digestion. Several possible explanations for this apparent discrepancy between reducing sugar release and unchanged MW profiles should be considered. First, the degradation might occur predominantly on side chains rather than the main chain. A previous study on enzymatic hydrolysis of pectin demonstrated that polysaccharide MW changes were sensitive to the branch degradation, while a little change in MW was observed for an acid-extracted pectin from sugar beet pulp (ABP) after branch degradation, which could be attributed to its low branch content (arabinose < 7%) [42]. In this study, EPS and IPS might also have limited branching, which led to a negligible change in their MW. Second, due to the limitation of HPGPC, any low-MW products (e.g., oligosaccharides or monosaccharides) would likely elute near the solvent peak or beyond the total column volume and thus would not appear as distinct new peaks in the MW profiles. Third, polysaccharides as macromolecules usually form aggregates in the aqueous solution to encapsulate their terminal ends. The digestion process might induce certain extension of the aggregates to expose the reduced ends so that they could be detected by DNS reagents.

In addition, regarding the increase in reducing sugar content without a significant change in MW profiles during SIF digestion, another two concepts should be further clarified. First, the DNS assay was sensitive to pH and matrix composition, and the relatively small increases after SIF digestion (approximately 2–4%) therefore might be attributed to the environmental factors or, alternatively, might indicate limited cleavage of glycosidic bonds rather than extensive degradation. Second, HPGPC analysis employed in this study assumed that polysaccharides behaved as individual, non-interacting chains in solution. However, for aggregated polysaccharides (EPS and IPS), the elution behavior might deviate substantially from ideal size-exclusion behavior. Digestion-induced disruption of aggregates without covalent bond cleavage could theoretically alter the HPGPC profile in ways that mimic molecular weight changes, or conversely, mask actual degradation. Based on all the above, both EPS and IPS were moderately affected by in vitro digestion. Any changes induced during digestion were likely confined to partial hydrolysis or alterations in conformation or aggregation state without extensive main-chain degradation.

### 3.7. Effects of EPS and IPS on Microbial Composition

Regarding the high resistance of EPS and IPS against in vitro digestion, fecal fermentation was further conducted to investigate their prebiotic potential with inulin as the prebiotic reference. Sobs index was applied to evaluate bacterial diversity and Shannon index was used to assess microbial richness, and both of them reflected the bacterial alpha diversity. According to Figure S4a,b and Table S10, EPS and IPS increased the bacterial alpha diversity compared to blank, while inulin decreased the alpha diversity. Inulin was reported to show strong bifidogenic effects by sharply increasing the abundance of *Bifidobacterium*, which compressed the growth of other bacterial species [16]. This led to a decline in bacterial diversity. This result was consistent with our previous report [16]. Principal coordinate analysis (PCoA) and sample level cluster were two primary methods for beta diversity analysis. The microbial structures were remarkably different in the EPS, IPS and inulin groups from blank (Figure S4c,d). The PCoA plots and sample level clusters of EPS and IPS were adjusted to each other but separated from that of inulin. This suggested that the three carbohydrates could modulate microbial composition, and the modulation was similar to the intervention of EPS and IPS but different from that with inulin. EPS and IPS had similar monosaccharide compositions (glucose, galactose and mannose) as well as functional groups, while inulin was mainly composed of fructose [43]. Therefore, the metabolic pathways of gut microbiota on EPS and IPS were likely similar, leading to similarities in the bacterial composition. There still existed distance in PCoA plots and sample level clusters between the EPS and IPS groups, indicating different bacterial profiles in these two groups. This might result from the different MW and fine structures (microstructures and aggregate state) of EPS and IPS.

**Figure 4:**
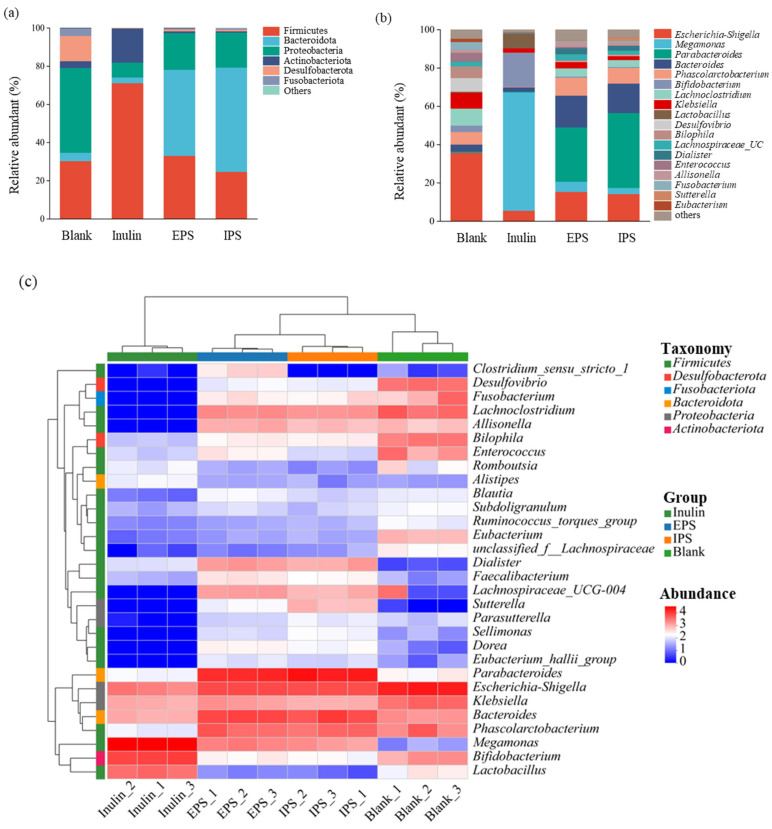
Microbial composition at (**a**) phylum and (**b**) genus level with the most abundant ones (>1%) and (**c**) the changes in bacteria at genus level.

Figure 4 presents the taxa relative abundance of fecal bacteria at the phylum and genus levels based on operational taxonomic unit (OTU). At the phylum level (Figure 4a and Table S11), *Proteobacteria* (44.23%), *Firmicutes* (30.19%), *Desulfobacterota* (13.18%), *Bacteroidota* (4.33%), *Fusobacteriota* (4.04%) and *Actinobacteriota* (3.56%) were mainly found in blank with total relative abundance over 99%, while *Desulfobacterota* and *Fusobacteriota* were nearly absent (<1%) in the three treated groups. The reduction in the abundance of *Desulfobacterota* was reported to show assistance in maintaining the intestinal barrier to reduce inflammatory response [44]. The increase in *Fusobacteriota* was considered to be associated with malnutrition [45]. The absence of this phylum indicated that EPS, IPS and inulin could be utilized by gut microbiota as carbon sources for their growth. *Proteobacteria* containing a large group of pathogenic bacteria was sharply compressed by inulin (7.76%) compared to blank. The less but significant decrease in *Proteobacteria* abundance was also observed in IPS (18.36%) and EPS (19.24%). The suppression of *Desulfobacterota*, *Fusobacteriota* and *Proteobacteria* indicated a more stable, less inflammatory gut microbial community. The abundance of *Actinobacteriota* decreased more significantly by IPS (0.64%) and less significantly by EPS (0.91%), while it increased more by inulin (17.94%) versus blank. Usually, *Actinobacteriota* in the intestines of healthy young adults is significantly higher than that in the elderly [46]. However, a recent study found that significant enrichment in *Actinobacteriota* was observed in the patient with diabetic foot and lower limb atherosclerosis [47]. Therefore, the changes in *Actinobacteriota* abundance could not be simply considered as beneficial or harmful. It should be interpreted in conjunction with specific factors such as age and health status of host. The growth of *Bacteroidota* was significantly upregulated by EPS and IPS with the higher abundance in the IPS group (54.57%) and the lower in the EPS group (45.08%). Some species from *Bacteroidota*-encoding starch-utilization system (Sus)-like system had a strong ability to degrade and use high MW polysaccharides for their growth [48]. Therefore, IPS with higher MW led to more significant changes in *Bacteroidota* than EPS. The ratio of *Firmicutes*/*Bacteroidota* (F/B) was decreased by IPS (0.45) and EPS (0.73) compared to blank (6.97). The ratio of F/B was commonly regarded to be closely associated with obesity and diabetes, and in the context of metabolic disorders, a lower F/B ratio contributed to reducing the risk of obesity [49].

At the genus level (Figure 4b and Table S12), *Megamonas*, *Dialister* and *Sutterella* were nearly absent in blank, while the abundance of the former two bacteria was increased by EPS, IPS and inulin, and the latter was only enhanced by EPS and IPS. Compared to the EPS group (3.23% for *Megamonas* and 0.37% for *Sutterella*), the IPS group exhibited significantly higher relative abundances of these two bacteria at 5.29% and 1.73%, respectively. The growth of *Megamonas* in gut would enhance the generation of short-chain fatty acids (SCFAs), especially acetate acid and propionic acid which were some of the most important beneficial metabolites generated by gut microbiota [50]. *Dialister* was one of the valerate producers in intestine and its growth was closely related to inflammation resolution [51,52]. A clinical report showed that after anti-diabetes treatment, the gut microbial diversity of the patients tended to recover with *Sutterella* increasing [53]. Compared to blank, the relative abundance of *Parabacteroides* and *Bacteroides* was sharply increased by IPS at 72.4 times and 4.2 times, respectively, and the increase in *Parabacteroides* was less significant by EPS at 52.6 times. *Parabacteroides* and *Bacteroides*, possessing multiple polysaccharide utilization loci (PULs) and carbohydrate-active enzymes (CAZymes), were two important polysaccharide degraders in gastrointestinal tract [54,55]. Some species from *Parabacteroides* such as *P. goldsteinii* were previously reported to show stronger degradation capability on *Cordyceps sinensis* polysaccharides than *Bacteroides* [55]. In addition, the growth of *Parabacteroides* was positively correlated with the galactose contents in carbon sources [55]. Therefore, in this study, *Parabacteroides* with a higher relative abundance than *Bacteroides* was observed with the intervention of EPS and IPS, and IPS with higher galactose content resulted in much more significant influence on *Parabacteroides* than EPS. The abundance of *Escherichia-Shigella*, which is kept in a low level in healthy hosts and would explode to be a dominant population when intestinal homeostasis is disrupted, was decreased in all treated groups compared to blank, with the lowest in the inulin group (5.54%) followed by the IPS group (14.22%) and EPS group (15.38%) [56]. Some other noteworthy shifts were observed at genus level as well (Figure 4c). The abundance of *Desulfovibrio*, *Enterococcus*, *Eubacterium*, and unclassified_f_*Lachnospiraceae* were significantly decreased in treated groups, while *Faecalibacterium* was increased by EPS and IPS versus blank. Remarkably, the relative abundance of *Clostridium*_*sensu*_*stricto*_1 was downregulated by IPS but upregulated by EPS. The changes in *Clostridium*_*sensu*_*stricto*_1 was closely related to alterations in nutrient utilization and energy metabolism [57]. Although EPS and IPS had comparable primary structures, their fine structures, especially microstructures and aggregate state, were distinguished. Therefore, some of the bacteria might have a different utilization selection of these two polysaccharides.

In general, both EPS and IPS had modulation effects on gut bacterial composition toward a healthier and more non-susceptible microecological balance. Most of the changes were similar to intervention of EPS and IPS due to the similar primary structures of these two polysaccharides. IPS with relatively higher MW resulted in much more significant shifts in bacterial composition. There also existed totally different growth trends in some bacteria resulting from the different fine structures of EPS and IPS.

### 3.8. Effects of EPS and IPS on Short-Chain Fatty Acids

Short-chain fatty acids (SCFAs), as the key beneficial metabolites derived from gut microbiota, play important roles in providing energy for intestinal cells, preserving intestinal barrier integrity, and regulating immune function [58,59]. Table 4 presents the production of six individual SCFAs and their sum after 48 h fermentation with intervention of different polysaccharide samples. Acetic acid, propionic acid and butyric acid were the major SCFAs with most of their concentrations higher than 1 mM, which was consistent with previous reports [60,61]. The total SCFA production was lowest in blank at 268.73 mM. The production of total SCFAs in treated groups was in the order of EPS (315.18 mM) > IPS (302.81 mM) > inulin (279.06 mM). This again confirmed that the three polysaccharides could be utilized by gut microbiota for their metabolism, and the bacteria preferred EPS mostly followed by IPS and inulin to produce SCFAs. Bacteria had different preferences on carbon sources with different monosaccharide compositions. Most of the gut bacteria could not directly use high-MW polysaccharides. Some species belonging to *Bacteroidota* and *Actinobacteriota* could encode various CAZymes to hydrolyze complex carbohydrate polymers and the other bacteria would only consume the hydrolysates through cross feeding [54,62]. Therefore, carbon sources with lower MW might be preferentially utilized by most gut bacteria. The production of acetic acid, propionic acid, butyric acid and valeric acid was significantly promoted by EPS and IPS, while a lower concentration of *i*-butyric acid and *i*-valeric acid was detected in these two groups compared to blank. Both *i*-butyric acid and *i*-valeric acid are mainly generated from fermentation on proteins. Saccharolytic fermentation was a more cost-effective process than proteolytic fermentation to provide energy, and the bacteria had priorities to use saccharides than proteins when sufficient polysaccharides were in the gut environment [63]. Thus, the reduction in the production of *i*-butyric acid and *i*-valeric acid might be attributed to the reduced reliance of microbiota on proteins as a carbon source.

### 3.9. Bacterial Metabolomics

**Figure 5:**
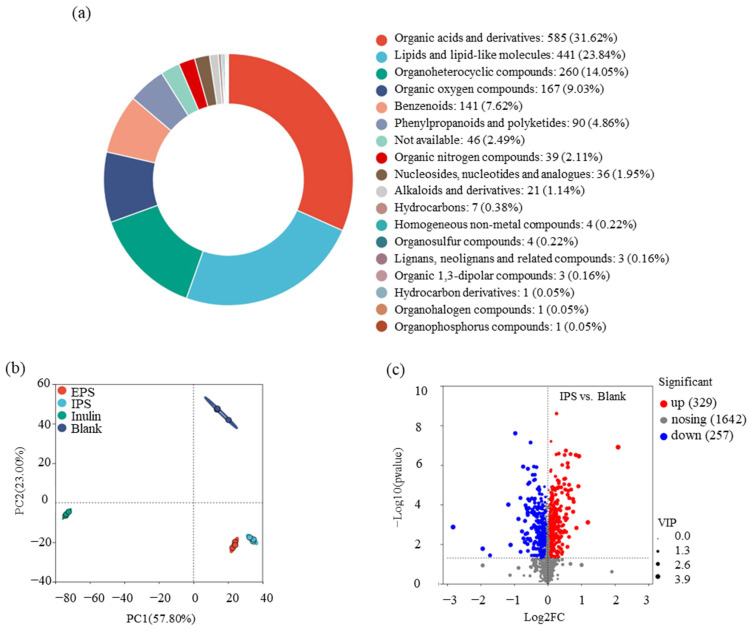

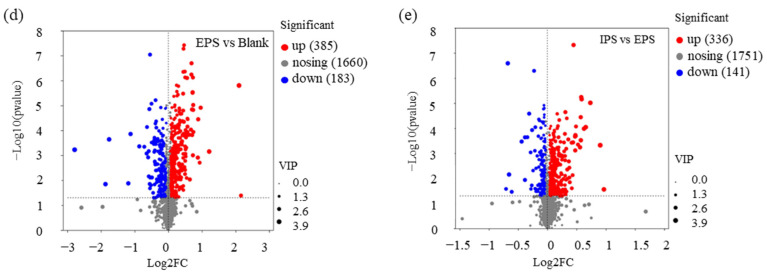
(**a**) Metabolite categories based on the human metabolome database (HMDB), (**b**) PCoA analysis of metabolic profiles and (**c**–**e**) metabolite volcano plots among blank, IPS group and EPS group.

To further explore how EPS and IPS influence gut bacterial metabolism, the metabolic profiles of gut microbiota were analyzed. In total, 2228 metabolic compounds were detected and annotated into 14 primary categories according to the human metabolome database (HMDB) (Figure 5a). The PCoA plot showed a clear clustering separation across all groups (Figure 5b), suggesting that bacterial metabolites differed between each of the two groups. Differential metabolites were screened based on the standards of variable importance for projection (VIP) value > 1.0, *p*-value < 0.05 and foldchange (FC) value > 1 or <1 (Figure 5c–e). There were 586 differential metabolic compounds between IPS group and blank, 568 between EPS group and blank, and 477 between IPS group and EPS group. This indicated less significant changes in bacterial metabolites between the EPS group and IPS group compared to these two polysaccharide groups versus blank. The top differential metabolites belonged to vitamin, amino acids, purine or their derivatives such as chenodeoxycholylarginine, 25-hydroxyvitamin D2 and guanine, whose contents were upregulated by EPS and/or IPS (Table S13). It should be noted that most of the remaining differential metabolites had limited records regarding their bioactive functions, and their biological relevance remained to be elucidated. In contrast, a small subset of the identified metabolites had been previously reported to confer beneficial effects, including spermidine, gallic acid, and indolelactic acid, all of which were also increased by EPS and/or IPS (Table S13). Spermidine, a natural polyamine, showed great potential in anti-aging and protecting the cardiovascular and nervous system [64]. Gallic acid was an organic acid belonging to polyphenolic compounds with antioxidant and anti-inflammatory effects [65]. Indolelactic acid, a key product of tryptophan metabolism, served as a ligand for both the hydroxycarboxylic acid receptor and the aryl hydrocarbon receptor to take part in maintaining intestinal and systemic immune homeostasis [66].

**Figure 6:**
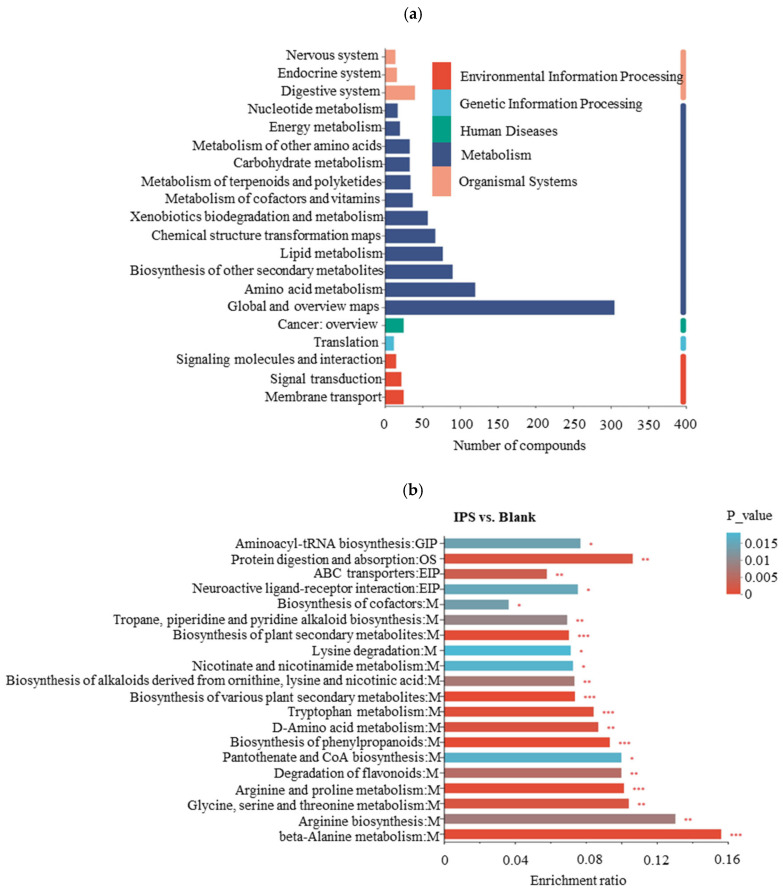

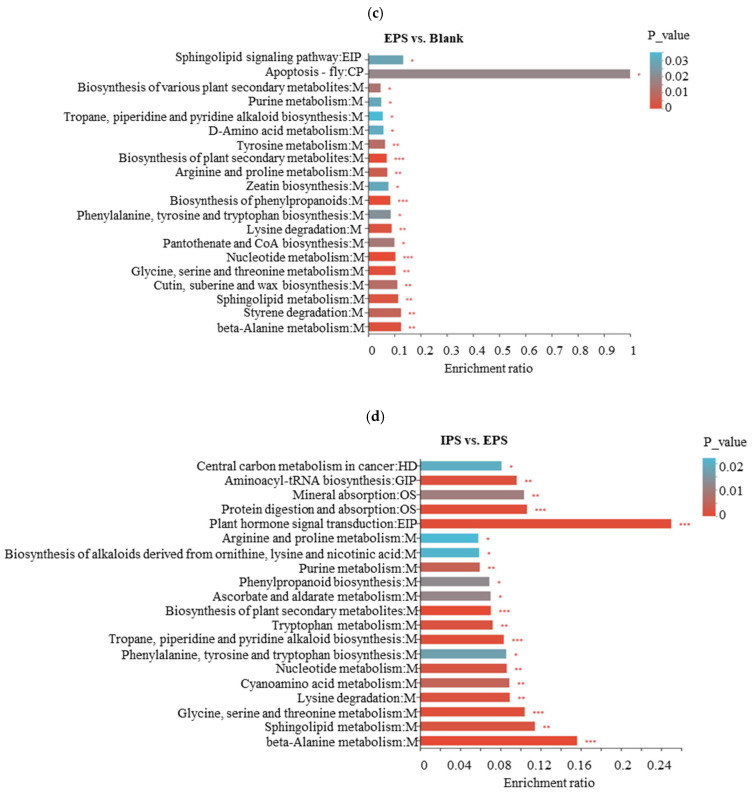
Metabolite categories (**a**) and differential metabolite analysis among blank, EPS group and IPS group (**b**–**d**) based on KEGG pathway. *, *p* < 0.05; **, *p* < 0.01; ***, *p* < 0.001, *n* = 3.

The microbial metabolites were further analyzed based on KEGG pathway. The identified metabolic compounds were classified into five primary pathway groups including environmental information processing, genetic information processing, human diseases, metabolism and organismal system (Figure 6a). Compared to blank, EPS and IPS mainly induced significant changes in the pathway group of metabolism (Figure 6b–d). This suggested that both EPS and IPS would regulate the gut microbiota on their metabolic pathway rather than directly exhibiting pathological or medicinal effects. This finding aligned with our earlier study that polysaccharides extracted from *Rosa roxburghii* Tratt pomace would alter the fecal bacterial metabolites mainly in the group of metabolism [14]. Several studies investigating polysaccharides modulating gut microbiota also had similar results even on disease models. *Lycium barbarum* polysaccharide (LBP) were reported to improve the ovarian reserve by modification on gut bacterial composition and metabolism, and the changes in bacterial metabolites were mainly found in the arginine biosynthesis, glycerophospholipid metabolism and steroid hormone biosynthesis pathways [67]. An in vivo study found that *Pueraria lobata* polysaccharide PLP-3 would ameliorate ischemic brain injury by improving the intestinal metabolites belonging to amino acid biosynthesis pathway [68].

### 3.10. Correlation Between Bacteria and Metabolites

**Figure 7:**
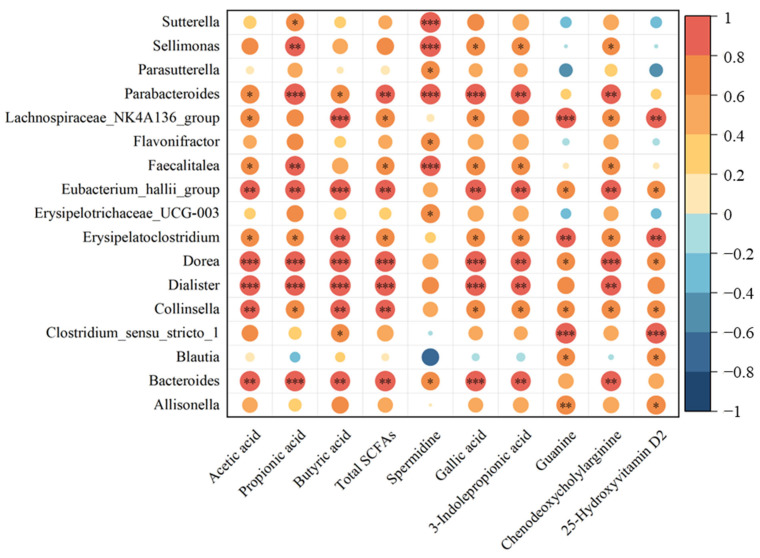
The relationship between differential metabolites and the most abundant bacteria based on Spearman correlation. *, *p* < 0.05; **, *p* < 0.01; ***, *p* < 0.001.

The relationship between the most abundant bacteria and several beneficial metabolites including SCFAs was further analyzed to explore the effects of EPS and IPS on gut microbiota using Spearman correlation (Figure 7). The metabolites were significantly positively regulated by most of the bacteria including *Bacteroides*, *Dorea*, *Lachnospiraceae*_NK4A136_group and *Faecalitalea.* These bacteria were commensal gut microorganisms, and they often exhibit synergistic rather than competitive relationships under normal conditions. Therefore, when the overall gut environment was conducive for proliferation, bacteria trended to grow, leading to the increase in metabolites. Guanine was negatively regulated by several species such as *Parasutterella*, *Sutterella* and *Erysipelotrichaceae*_UGG-003. Some *Parasutterella* species (e.g., *Parasutterella excrementihominis*) possessed purine metabolic pathways and could utilize guanine as a nitrogen source [69,70]. Thus, the increase in the abundance of *Parasutterella* might result in the decrease in guanine. The regulation of 25-hydroxyvitamin D2 had a similar trend to that of guanine by the gut flora. These results suggested the intervention of EPS and IPS enhanced the correlation between the microbiota and their beneficial metabolites.

Structure–activity relationship of polysaccharides has always been focused. Most of the recent studies paid attention to the MW of polysaccharides. Wu et al. reported that pectic polysaccharides with higher MW had more significant benefits on bacterial composition and metabolism [71], while Hou et al. found that *Astragalus* polysaccharides fractions with MW less than 10 kDa exhibited more significant effects to promote beneficial bacterial growth and enhance SCFA production [72]. In this study, IPS with higher proportion of high-MW fractions had stronger modulation on bacterial composition, EPS with lower MW promoted higher SCFA production, and EPS and IPS led to entirely different metabolic profiles. Therefore, it could not be simply assumed that polysaccharides with a high or low MW were better for host health. The utilization of polysaccharide-modulating gut microbiota should depend on the specific target. In addition, it is worth noting that certain bacteria species exhibited entirely different growth trends, which was highly probably due to the distinct fine structures (aggregate state and microstructures) of EPS and IPS. This indicated that besides MW, the fine structures of polysaccharides also had profound impact on the bacterial population.

## 4. Conclusions

The liquid medium composition of *Cordyceps farinosa* Cf-GZU06 was optimized and the resulting mycelial biomass was 44.797 g/L which was 1.9 times that obtained from the unoptimized basal medium. The high yield suggested *C. farinosa* Cf-GZU06 as a potential alternative for *C. sinensis* and *C. militaris.* Intracellular (IPS) and extracellular polysaccharides (EPS) were obtained from *C. farinosa* Cf-GZU06 mycelia and its fermentation broth, respectively. EPS and IPS shared similar monosaccharide compositions and nearly identical FT-IR profiles, suggesting comparable primary structures. The differences in their aggregate states and microstructures indicated that their fine structures differed. Both of these two polysaccharides showed limited susceptibility in in vitro digestion with less than 17% (*w*/*w*) of reducing sugar detected and no substantial shift in their MW profiles during the digestion process. EPS and IPS could regulate the gut microbiota by increasing the abundance of some beneficial bacterial species and significantly promoting the production of SCFAs. The microbial metabolic files were also significantly altered with the intervention of EPS and IPS. Regarding the high resistance to digestion and the modulation on gut microbiota in bacterial composition and metabolites, EPS and IPS had strong prebiotic effects and could be applied as prebiotic candidates. Based on the structure–prebiotic activity relationship of these two polysaccharides, IPS with higher MW was recommended for applications targeting gut bacterial composition modulation, whereas EPS with lower MW was more suitable for promoting SCFA production. For other metabolic outcomes, the choice between IPS and EPS should be tailored to the specific metabolic endpoints of interest. However, in this study, both EPS and IPS were a mixture of several fractions differing in molecular weight, and the current study only conducted a preliminary investigation into their general structures. Future work in purification and detailed structural characterization of EPS and IPS is of great necessity to reveal the structure–activity relationship of the polysaccharides. In addition, all the findings in the present study were based on in vitro assay and the correlation between bacteria and their metabolites were only dependent on statistical analysis. Further investigation involving animal models and mechanistic support by experimental validation is necessary to confirm the prebiotic effects of EPS and IPS.

## Figures and Tables

**Table 1:** Yield and major chemical content and physical property of EPS and IPS.

	EPS	IPS
Yield	0.60 g/L	1.63% (*w*/*w*)
Carbohydrate content (%, *w*/*w*)	75.86 ± 0.93	61.94 ± 0.82
Protein content (%, *w*/*w*)	1.88 ± 0.09	4.60 ± 0.11
Moisture content (%, *w*/*w*)	9.24 ± 0.09	8.40 ± 0.09
Average particle size (nm)	846.75 ± 20.58 128.60 ± 26.02	61.19 ± 2.99
Zeta potential (mV)	−8.27 ± 0.11	−2.71 ± 0.09

**Table 2:** The major peaks in HPGPC profiles of EPS and IPS.

	Retention Time (min)	Molecular Weight (kDa)	Area (%)
EPS	29.363	25.46	100.00
IPS	26.827	136.82	85.62
	29.707	22.96	14.38

**Table 3:** Reducing sugar content of EPS and IPS during in vitro digestion.

Digestion Time (min)	Reducing Sugar Content (%, *w*/*w*)	
	EPS	IPS
SSF		
0	2.47 ± 0.14 ^a^	2.60 ± 0.08 ^a^
5	2.38 ± 0.07 ^a^	2.57 ± 0.10 ^a^
SGF		
0	5.56 ± 0.22 ^c^	5.66 ± 0.17 ^c^
60	4.51 ± 0.43 ^b^	5.07 ± 0.22 ^b^
120	4.96 ± 0.18 ^b^	4.96 ± 0.15 ^b^
SIF		
0	11.16 ± 0.30 ^d^	11.94 ± 0.45 ^d^
60	12.30 ± 0.11 ^e^	14.42 ± 0.29 ^e^
120	13.86 ± 0.30 ^f^	16.10 ± 0.16 ^f^

SSF: Simulated saliva digestion fluid; SGF: simulated gastric digestion fluid; SIF: simulated intestinal digestion fluid; a, b, c, d, e, f: different letters indicate significant difference (*p* < 0.05) among different digestion duration for the same polysaccharide sample, ANOVA, *n* = 3.

**Table 4:** Short-chain fatty acids (SCFAs) production with interference of different polysaccharides.

mM	Acetic Acid	Propionic Acid	Butyric Acid	*i*-Butyric Acid	Valeric Acid	*i*-Valeric Acid	Total SCFAs
Blank	262.94 ± 5.71 ^b^	2.78 ± 0.04 ^d^	1.36 ± 0.07 ^c^	0.68 ± 0.10 ^a^	0.30 ± 0.01 ^b^	0.68 ± 0.22 ^a^	268.73 ± 6.16 ^c^
Inulin	273.13 ± 9.47 ^b^	5.52 ± 0.12 ^c^	0.41 ± 0.01 ^d^	ND	ND	ND	279.06 ± 9.57 ^c^
EPS	299.99 ± 9.66 ^a^	9.23 ± 0.21 ^b^	4.68 ± 0.06 ^a^	0.39 ± 0.01 ^c^	0.47 ± 0.01 ^a^	0.42 ± 0.02 ^c^	315.18 ± 9.49 ^a^
IPS	288.05 ± 3.03 ^a^	9.96 ± 0.10 ^a^	3.38 ± 0.25 ^b^	0.46 ± 0.02 ^b^	0.50 ± 0.04 ^a^	0.46 ± 0.01 ^b^	302.81 ± 2.81 ^b^

a, b, c, d: Different letters indicate significant difference (*p* < 0.05) in the short-chain fatty acid production among different groups, ANOVA, *n* = 3. ND: not detectable.

## Data Availability

The original contributions presented in the study are included in the article/Supplementary Materials, further inquiries can be directed to the corresponding author.

## Supplementary Materials

The following supporting information can be downloaded at https://www.mdpi.com/article/10.3390/foods15112038/s1.

## References

[1] Zheng P., Xia Y., Zhang S., Wang C. (2013). Genetics of Cordyceps and related fungi. Appl. Microbiol. Biotechnol..

[2] Olatunji O.J., Tang J., Tola A., Auberon F., Oluwaniyi O., Ouyang Z. (2018). The genus Cordyceps: An extensive review of its traditional uses, phytochemistry and pharmacology. Fitoterapia.

[3] Nxumalo W., Elateeq A.A., Sun Y. (2020). Can Cordyceps cicadae be used as an alternative to Cordyceps militaris and Cordyceps sinensis?—A review. J. Ethnopharmacol..

[4] Cordyceps Militaris Market Forecasts to 2032—Global Analysis. https://www.giiresearch.com/report/smrc1813219-cordyceps-militaris-market-forecasts-global.html.

[5] Tong C., Wei J., Pan G., Li C., Zhou Z. (2022). Study of pathogenesis using fluorescent strain of Cordyceps farinosa revealed infection of Thitarodes armoricanus larvae via digestive tract. Insects.

[6] Tong C., Li T., Luo S., Chen R., Chen S., Wei J., Qing Y., Qin S., Pan G., Li C. (2023). Detection of the pathogenic fungus Cordyceps farinosa in the Thitarodes armoricanus soil-rearing environment based on nucleic acid targets. Can. J. Microbiol..

[7] An G.-H., Han J.-G., Park H.-S., Sung G.-H., Kim O.-T. (2021). Identification of an oxidosqualene cyclase gene involved in steroidal triterpenoid biosynthesis in Cordyceps farinosa. Genes.

[8] Krawczyk-Łebek A., Dymarska M., Janeczko T., Kostrzewa-Susłow E. (2021). New glycosylated dihydrochalcones obtained by biotransformation of 2′-hydroxy-2-methylchalcone in cultures of entomopathogenic filamentous fungi. Int. J. Mol. Sci..

[9] Chen L., Liu X., Zheng K., Wang Y., Li M., Zhang Y., Cui Y., Deng S., Liu S., Zhang G. (2024). Cordyceps polysaccharides: A review of their immunomodulatory effects. Molecules.

[10] Yao L., Zhu L., Chen C., Wang X., Zhang A., Gao S., Wu J., Qin L. (2024). A systematic review on polysaccharides from fermented Cordyceps sinensis: Advances in the preparation, structural characterization, bioactivities, structure-activity relationships. Int. J. Biol. Macromol..

[11] Akaki J., Matsui Y., Kojima H., Nakajima S., Kamei K., Tamesada M. (2009). Structural analysis of monocyte activation constituents in cultured mycelia of Cordyceps sinensis. Fitoterapia.

[12] Rong L., Li G., Zhang Y., Xiao Y., Qiao Y., Yang M., Wei L., Bi H., Gao T. (2021). Structure and immunomodulatory activity of a water-soluble α-glucan from Hirsutella sinensis mycelia. Int. J. Biol. Macromol..

[13] Li L.-Q., Song A.-X., Wong W.-T., Wu J.-Y. (2021). Isolation and assessment of a highly-active anti-inflammatory exopolysaccharide from mycelial fermentation of a medicinal fungus Cs-HK1. Int. J. Mol. Sci..

[14] Chen Z.-H., Yuan X.-h., Tu T.-T., Wang L., Mao Y.-H., Luo Y., Qiu S.-Y., Song A.-X. (2024). Characterization and prebiotic potential of polysaccharides from Rosa roxburghii Tratt pomace by ultrasound-assisted extraction. Int. J. Biol. Macromol..

[15] Tu T.-T., Yuan X.-H., Mao Y.-H., Wang Y.-C., Chen Z.-H., Wang L., Luo Y., Wang C.-X., Qiu S.-Y., Song A.-X. (2025). Characterization of two pectic polysaccharides from Rosa roxburghii Tratt fruit and their protection on Bifidobacterium against antibiotic damage. Food Biosci..

[16] Yuan X.-H., Tu T.-T., Mao Y.-H., Wang Y.-C., Huang M.-Q., Wang L., Luo Y., Wang C.-X., Qiu S.-Y., Deng B. (2026). Characterization and bifidogenic effects of a low-molecular weight polysaccharide isolated from a Chinese herb, Polygonatum kingianum Coll. et Hemsl (Huangjing) rhizome. Carbohydr. Polym..

[17] Weiss G.A., Hennet T. (2017). Mechanisms and consequences of intestinal dysbiosis. Cell. Mol. Life Sci. CMLS.

[18] Magne F., Gotteland M., Gauthier L., Zazueta A., Pesoa S., Navarrete P., Balamurugan R. (2020). The Firmicutes/Bacteroidetes Ratio: A Relevant Marker of Gut Dysbiosis in Obese Patients?. Nutrients.

[19] Prebiotics Market Size, Share & Industry Analysis, By Product Type (Inulin, Fructo-oligosaccharides (FOS), Galacto-oligosaccharides (GOS), Others), By Applications (Food and Beverage, Pharmaceutical, Animal Feed), Others and Regional Forecast, 2026–2034. https://www.fortunebusinessinsights.com/prebiotics-market-102286.

[20] Yuan Q., Xie F., Tan J., Yuan Y., Mei H., Zheng Y., Sheng R. (2022). Extraction, structure and pharmacological effects of the polysaccharides from Cordyceps sinensis: A review. J. Funct. Foods.

[21] Mao Y.-H., Song A.-X., Li L.-Q., Yang Y., Yao Z.-P., Wu J.-Y. (2020). A high-molecular weight exopolysaccharide from the Cs-HK1 fungus: Ultrasonic degradation, characterization and in vitro fecal fermentation. Carbohydr. Polym..

[22] Chen S., Wang J., Fang Q., Dong N., Fang Q., Cui S.W., Nie S. (2021). A polysaccharide from natural Cordyceps sinensis regulates the intestinal immunity and gut microbiota in mice with cyclophosphamide-induced intestinal injury. Food Funct..

[23] Song A.-X., Mao Y.-H., Siu K.-C., Wu J.-Y. (2018). Bifidogenic effects of Cordyceps sinensis fungal exopolysaccharide and konjac glucomannan after ultrasound and acid degradation. Int. J. Biol. Macromol..

[24] Li Q.-Z., Xiong C., Wong W.C., Zhou L.-W. (2024). Medium composition optimization and characterization of polysaccharides extracted from Ganoderma boninense along with antioxidant activity. Int. J. Biol. Macromol..

[25] Song A.-X., Li L.-Q., Yin J.-Y., Chiou J.-C., Wu J.-Y. (2020). Mechanistic insights into the structure-dependant and strain-specific utilization of wheat arabinoxylan by Bifidobacterium longum. Carbohydr. Polym..

[26] Brodkorb A., Egger L., Alminger M., Alvito P., Assunção R., Ballance S., Bohn T., Bourlieu-Lacanal C., Boutrou R., Carrière F. (2019). INFOGEST static in vitro simulation of gastrointestinal food digestion. Nat. Protoc..

[27] Miller G.L. (1959). Use of dinitrosalicylic acid reagent for determination of reducing sugar. Anal. Chem..

[28] Xie M., Chen W., Lai X., Dai H., Sun H., Zhou X., Chen T. (2019). Metabolic responses and their correlations with phytochelatins in Amaranthus hypochondriacus under cadmium stress. Environ. Pollut..

[29] Leung P.H., Wu J.Y. (2007). Effects of ammonium feeding on the production of bioactive metabolites (cordycepin and exopolysaccharides) in mycelial culture of a Cordyceps sinensis fungus. J. Appl. Microbiol..

[30] Kim S.W., Hwang H.J., Xu C.P., Sung J.M., Choi J.W., Yun J.W. (2003). Optimization of submerged culture process for the production of mycelial biomass and exo-polysaccharides by Cordyceps militaris C738. J. Appl. Microbiol..

[31] Song L., Yang J., Kong W., Liu Y., Liu S., Su L. (2023). Cordyceps militaris polysaccharide alleviates ovalbumin-induced allergic asthma through the Nrf2/HO-1 and NF-κB signaling pathways and regulates the gut microbiota. Int. J. Biol. Macromol..

[32] Wang Q., Wood P.J., Cui W., Ross-Murphy S.B. (2001). The effect of autoclaving on the dispersibility and stability of three neutral polysaccharides in dilute aqueous solutions. Carbohydr. Polym..

[33] Song A.-X., Mao Y.-H., Siu K.-C., Tai W.C.S., Wu J.-Y. (2019). Protective effects of exopolysaccharide of a medicinal fungus on probiotic bacteria during cold storage and simulated gastrointestinal conditions. Int. J. Biol. Macromol..

[34] Hu T., Cai W., Cai W., Zheng Z., Xiao Y., Huang Q. (2021). Structure, size and aggregated morphology of a β-D-glucan from Lignosus rhinocerotis as affected by ultrasound. Carbohydr. Polym..

[35] Kim S.D. (2010). Isolation, structure and cholesterol esterase inhibitory activity of a polysaccharide, PS-A, from Cordyceps sinensis. J. Korean Soc. Appl. Biol. Chem..

[36] Sheng L., Chen J., Li J., Zhang W. (2011). An exopolysaccharide from cultivated Cordyceps sinensis and its effects on cytokine expressions of immunocytes. Appl. Biochem. Biotechnol..

[37] Zha Z., Zhang Z., Wei W., Nie W., Chu W., Huang F., Yue L., Wang S.-Y., Yin H. (2020). Isolation, structural characterization of polysaccharide from Cephalosporium sinensis mycelia and its anti-nephritic effects in adenine-induced CKD rats. Int. J. Biol. Macromol..

[38] Zhang Y., Lei Y., Qi S., Fan M., Zheng S., Huang Q., Lu X. (2023). Ultrasonic-microwave-assisted extraction for enhancing antioxidant activity of Dictyophora indusiata polysaccharides: The difference mechanisms between single and combined assisted extraction. Ultrason. Sonochem..

[39] Deng C., Sun Y., Fu H., Zhang S., Chen J., Xu X. (2016). Antioxidant and immunostimulatory activities of polysaccharides extracted from Tremella aurantialba mycelia. Mol. Med. Rep..

[40] Pan Y., Liu C., Jiang S., Guan L., Liu X., Wen L. (2024). Ultrasonic-assisted extraction of a low molecular weight polysaccharide from Nostoc commune Vaucher and its structural characterization and immunomodulatory activity. Ultrason. Sonochem..

[41] Du R., Guo W., Shen Y., Dai J., Zhang H., Fu M., Wang X. (2023). In situ assay of the reducing sugars in hydrophilic natural deep eutectic solvents by a modified DNS method. J. Mol. Liq..

[42] Oosterveld A., Beldman G., Voragen A.G. (2002). Enzymatic modification of pectic polysaccharides obtained from sugar beet pulp. Carbohydr. Polym..

[43] Mensink M.A., Frijlink H.W., van der Voort Maarschalk K., Hinrichs W.L.J. (2015). Inulin, a flexible oligosaccharide I: Review of its physicochemical characteristics. Carbohydr. Polym..

[44] Zhao Q., Hao Y., Yang X., Mao J., Tian F., Gao Y., Tian X., Yan X., Qiu Y. (2023). Mitigation of maternal fecal microbiota transplantation on neurobehavioral deficits of offspring rats prenatally exposed to arsenic: Role of microbiota-gut-brain axis. J. Hazard. Mater..

[45] Chibuye M., Mende D.R., Spijker R., Simuyandi M., Luchen C.C., Bosomprah S., Chilengi R., Schultsz C., Harris V.C. (2024). Systematic review of associations between gut microbiome composition and stunting in under-five children. npj Biofilms Microbiomes.

[46] Li J., Si H., Du H., Guo H., Dai H., Xu S., Wan J. (2021). Comparison of gut microbiota structure and Actinobacteria abundances in healthy young adults and elderly subjects: A pilot study. BMC Microbiol..

[47] Zhou M., Zhang H., Zhang R., Wei T., Zhou X. (2025). Increased abundance of actinobacteria and upregulation of primary bile acid biosynthesis in diabetic foot ulcers. Front. Cell. Infect. Microbiol..

[48] Wong J.P.H., Chillier N., Fischer-Stettler M., Zeeman S.C., Battin T.J., Persat A. (2024). Bacteroides thetaiotaomicron metabolic activity decreases with polysaccharide molecular weight. mBio.

[49] Karačić A., Renko I., Krznarić Ž., Klobučar S., Liberati Pršo A.-M. (2024). The association between the Firmicutes/Bacteroidetes ratio and body mass among European population with the highest proportion of adults with obesity: An observational follow-up study from Croatia. Biomedicines.

[50] Fusco W., Lorenzo M.B., Cintoni M., Porcari S., Rinninella E., Kaitsas F., Lener E., Mele M.C., Gasbarrini A., Collado M.C. (2023). Short-chain fatty-acid-producing bacteria: Key components of the human gut microbiota. Nutrients.

[51] Taylor H., Serrano-Contreras J.I., McDonald J.A., Epstein J., Fell J., Seoane R.C., Li J.V., Marchesi J.R., Hart A.L. (2020). Multiomic features associated with mucosal healing and inflammation in paediatric Crohn’s disease. Aliment. Pharmacol. Ther..

[52] Taylor H., McDonald J., Serrano Contreras J., Li J., Marchesi J., Hart A. (2020). DOP08 Deep remission in paediatric Crohn’s disease is associated with increased abundance of dialister species and increased valerate. J. Crohn’s Colitis.

[53] Gaike A.H., Paul D., Bhute S., Dhotre D.P., Pande P., Upadhyaya S., Reddy Y., Sampath R., Ghosh D., Chandraprabha D. (2020). The gut microbial diversity of newly diagnosed diabetics but not of prediabetics is significantly different from that of healthy nondiabetics. mSystems.

[54] Ndeh D., Gilbert H.J. (2018). Biochemistry of complex glycan depolymerisation by the human gut microbiota. FEMS Microbiol. Rev..

[55] Qu Z., Liu H., Yang J., Zheng L., Huang J., Wang Z., Xie C., Zuo W., Xia X., Sun L. (2025). Selective utilization of medicinal polysaccharides by human gut Bacteroides and Parabacteroides species. Nat. Commun..

[56] Zeng M.Y., Inohara N., Nuñez G. (2017). Mechanisms of inflammation-driven bacterial dysbiosis in the gut. Mucosal Immunol..

[57] Pei Y., Chen C., Mu Y., Yang Y., Feng Z., Li B., Li H., Li K. (2021). Integrated microbiome and metabolome analysis reveals a positive change in the intestinal environment of myostatin edited large white pigs. Front. Microbiol..

[58] Zhang D., Jian Y.-P., Zhang Y.-N., Li Y., Gu L.-T., Sun H.-H., Liu M.-D., Zhou H.-L., Wang Y.-S., Xu Z.-X. (2023). Short-chain fatty acids in diseases. Cell Commun. Signal..

[59] Ney L.-M., Wipplinger M., Grossmann M., Engert N., Wegner V.D., Mosig A.S. (2023). Short chain fatty acids: Key regulators of the local and systemic immune response in inflammatory diseases and infections. Open Biol..

[60] Xiong R.G., Zhou D.D., Wu S.X., Huang S.Y., Saimaiti A., Yang Z.J., Shang A., Zhao C.N., Gan R.Y., Li H.B. (2022). Health benefits and side effects of short-chain fatty acids. Foods.

[61] O’Riordan K.J., Collins M.K., Moloney G.M., Knox E.G., Aburto M.R., Fülling C., Morley S.J., Clarke G., Schellekens H., Cryan J.F. (2022). Short chain fatty acids: Microbial metabolites for gut-brain axis signalling. Mol. Cell. Endocrinol..

[62] Briggs J.A., Grondin J.M., Brumer H. (2021). Communal living: Glycan utilization by the human gut microbiota. Environ. Microbiol..

[63] Jardon K.M., Canfora E.E., Goossens G.H., Blaak E.E. (2022). Dietary macronutrients and the gut microbiome: A precision nutrition approach to improve cardiometabolic health. Gut.

[64] Ni Y.Q., Liu Y.S. (2021). New insights into the roles and mechanisms of spermidine in aging and age-related diseases. Aging Dis..

[65] Bai J., Zhang Y., Tang C., Hou Y., Ai X., Chen X., Zhang Y., Wang X., Meng X. (2021). Gallic acid: Pharmacological activities and molecular mechanisms involved in inflammation-related diseases. Biomed. Pharmacother..

[66] Yong C.C., Sakurai T., Kaneko H., Horigome A., Mitsuyama E., Nakajima A., Katoh T., Sakanaka M., Abe T., Xiao J.-Z. (2024). Human gut-associated Bifidobacterium species salvage exogenous indole, a uremic toxin precursor, to synthesize indole-3-lactic acid via tryptophan. Gut Microbes.

[67] Zheng H., Liang X., Zhou H., Zhou T., Liu X., Duan J., Duan J.-a., Zhu Y. (2023). Integrated gut microbiota and fecal metabolome analyses of the effect of Lycium barbarum polysaccharide on D-galactose-induced premature ovarian insufficiency. Food Funct..

[68] Zhang Y., Dou Z., Li S., Zhang H., Zeng S., Zuo X., Xiao Y., Zhang L., Li Z., Zhu Q. (2025). An ultrasonic degraded polysaccharide extracted from Pueraria lobata ameliorate ischemic brain injury in mice by regulating the gut microbiota and LPS-TLR4 pathway. Ultrason. Sonochem..

[69] Halimulati M., Wang R., Aihemaitijiang S., Huang X., Ye C., Zhang Z., Li L., Zhu W., Zhang Z., He L. (2023). Anti-hyperuricemic effect of anserine based on the gut-kidney axis: Integrated analysis of metagenomics and metabolomics. Nutrients.

[70] Ju T., Kong J.Y., Stothard P., Willing B.P. (2019). Defining the role of Parasutterella, a previously uncharacterized member of the core gut microbiota. ISME J..

[71] Wu J., Yu C., Shen S., Ren Y., Cheng H., Xiao H., Liu D., Chen S., Ye X., Chen J. (2023). RGI-type pectic polysaccharides modulate gut microbiota in a molecular weight-dependent manner in vitro. J. Agric. Food Chem..

[72] Huo Z., Li J., Li X., Xiao H., Lin Y., Ma Y., Li J., Yang H., Zhang C. (2024). Functional fractions of Astragalus polysaccharides as a potential prebiotic to alleviate ulcerative colitis. Int. J. Biol. Macromol..

