# Supplementary material for: Cordyceps farinosa Cf-GZU06: Mycelium Culture Medium Optimization and Polysaccharide Characterization with Prebiotic Effects
Source: Foods. 2026 Jun 5;15(11):2038. doi: 10.3390/foods15112038 (PMC13256718; doi:10.3390/foods15112038)
Supplement: Supplementary file 1 [file foods-15-02038-s001.zip › foods-4342004-supplementary/foods-4342004-supplementary.pdf]

Supplemental data

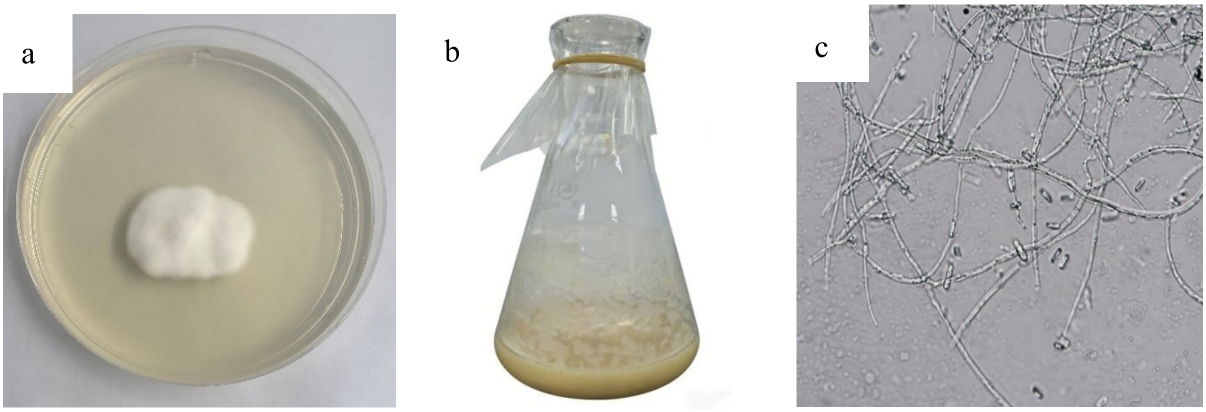

Figure S1. Morphological characteristics of *C. farinosa* Cf-GZU06 in solid and liquid cultures. (a) Mycelial colony on a perti dish, (b) mycelial culture in liquid medium and (c) mycelial morphology from liquid culture (40 ×).

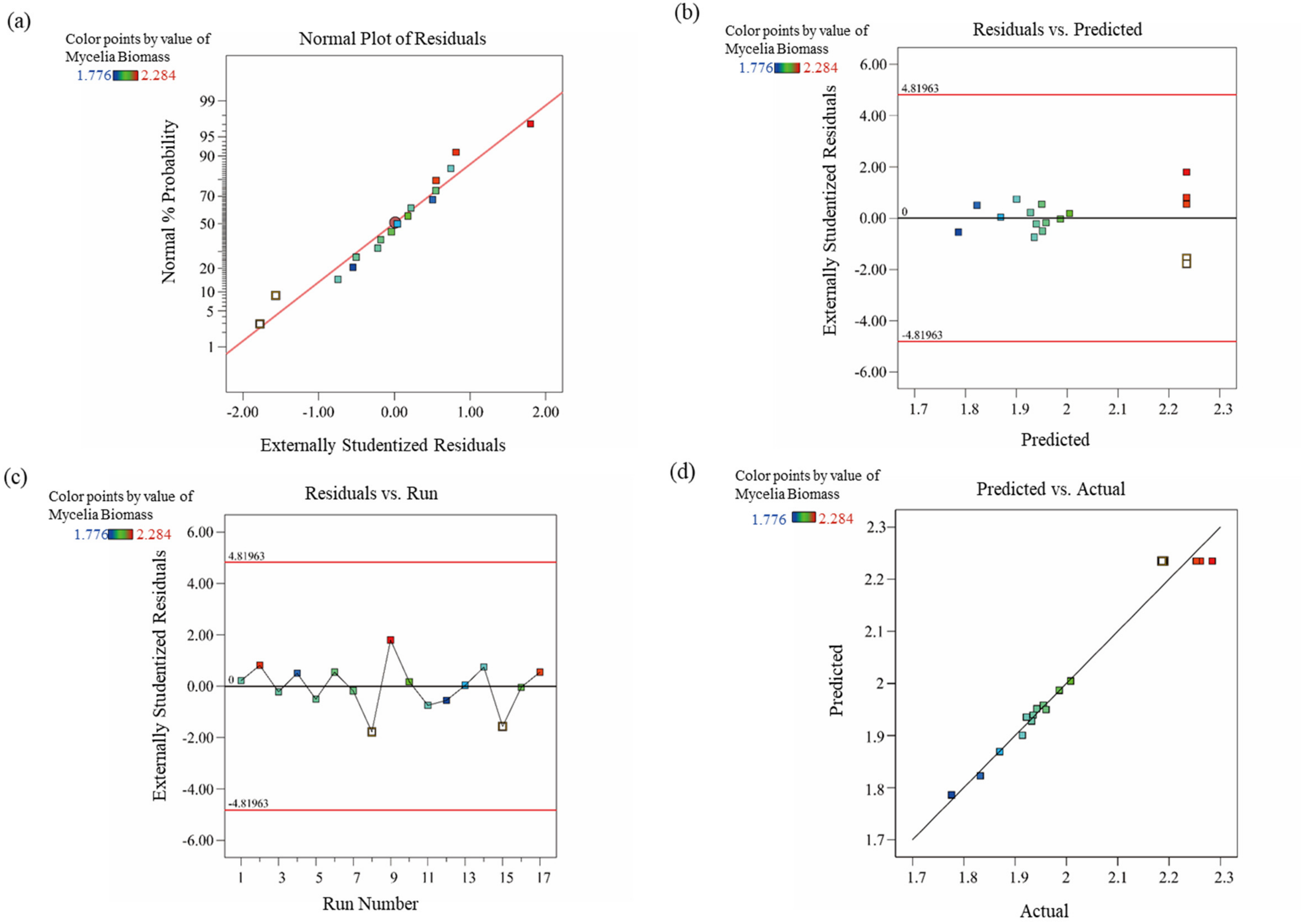

Figure S2. Diagnostic plots of regression model. (a) Normal probability plot of residuals, (b) Scatter plot of residuals versus predicted values, (c) Plot of residuals versus run order and (d) Plot of predicted values versus actual values.

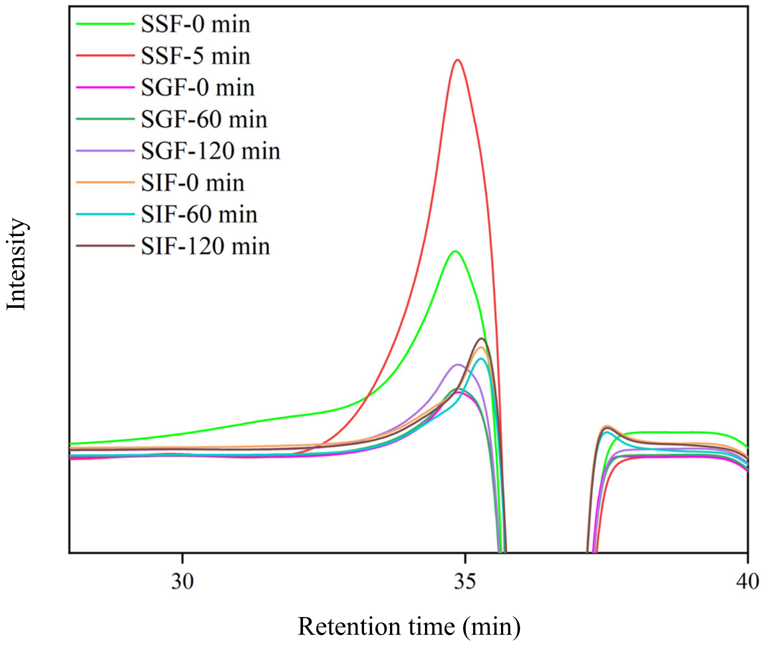

Figure S3. Molecular weight distribution changes of soluble starch during in vitro digestion. SSF, simulated saliva digestion fluid; SGF, simulated gastric digestion fluid; SIF, simulated intestinal digestion fluid.

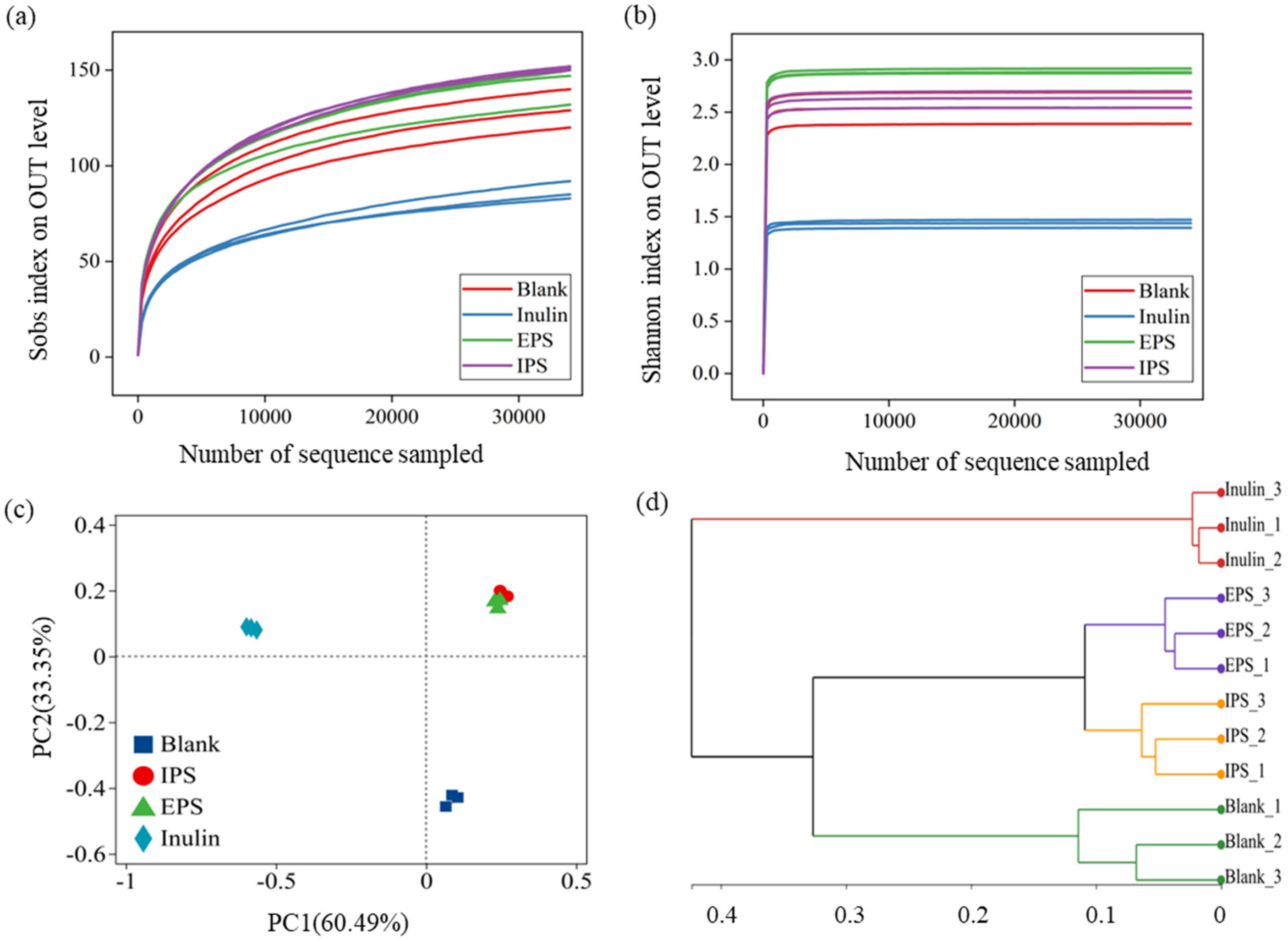

Figure S4. Microbial alpha diversity (a, b) and beta diversity (c, d) with the intervention of different polysaccharides.

Table S1. Factors and levels set for Plackett-Burman design.

Factor	Variable	Low level (-1)	High level (+1)
Culture time (d)	A	6	10
Glucose (g/L)	B	30	50
Peptone (g/L)	C	4	6
Yeast extract (g/L)	D	8	12
$MgSO_4 \cdot 7H_2O$ (g/L)	E	0.4	0.6
KH_2PO_4 (g/L)	F	0.6	1.4

Table S2. Experimental design and corresponding fungal biomass in Plackett-Burman design.

Run	Culture time (A, d)	Glucose (B, g/L)	Peptone (C, g/L)	Yeast extract (D, g/L)	$MgSO_4\cdot 7H_2O$ (E, g/L)	KH_2PO_4 (F, g/L)	Biomass (g/L)
1	10	50	4	12	0.6	1.4	27.480 ± 0.208
2	6	50	6	8	0.6	1.4	26.600 ± 0.185
3	10	30	6	12	0.4	1.4	21.260 ± 0.162
4	6	50	4	12	0.6	0.6	28.220 ± 0.196
5	6	30	6	8	0.6	1.4	19.980 ± 0.139
6	6	30	4	12	0.4	1.4	21.300 ± 0.150
7	10	30	4	8	0.6	0.6	17.780 ± 0.127
8	10	50	4	8	0.4	1.4	25.480 ± 0.173
9	10	50	6	8	0.4	0.6	25.420 ± 0.162
10	6	50	6	12	0.4	0.6	28.680 ± 0.208
11	10	30	6	12	0.6	0.6	21.020 ± 0.139
12	6	30	4	8	0.4	0.6	18.740 ± 0.127

Table S3. Analysis of the six variables in Plackett-Burman design based on fungal biomass

Source	Sum of squares	Degree of freedom	Mean square	*F*-value	*p*-value
Model	0.4253	6	0.0709	687.28	< 0.0001**
A: Culture time	0.0043	1	0.0043	42.12	0.0013**
B: Glucose	0.3730	1	0.3730	3617.03	< 0.0001**
C: Peptone	0.0025	1	0.0025	23.99	0.0045**
D: Yeast extract	0.0438	1	0.0438	424.39	< 0.0001**
E: $MgSO_4 \cdot 7H_2O$	0.0001	1	0.0001	0.95	0.3743
F: KH_2PO_4	0.0016	1	0.0016	15.23	0.0114*

*: Significance with $p < 0.05$.

**:Significance with $p < 0.01$.

Table S4. Experimental design and corresponding fungal biomass in the path of steepest ascent.

Run	Step size	Glucose (g/L)	Peptone (g/L)	Yest extract (g/L)	Biomass (g/L)
1	0	50	6	12	29.520 ± 0.173
2	0+1Δ	55	7	14	32.280 ± 0.196
3	0+2Δ	60	8	16	35.140 ± 0.196
4	0+3Δ	65	9	18	35.400 ± 0.208
5	0+4Δ	70	10	20	36.560 ± 0.219
6	0+5Δ	75	11	22	38.680 ± 0.231
7	0+6Δ	80	12	24	37.400 ± 0.219
8	0+7Δ	85	13	26	37.060 ± 0.208
9	0+8Δ	90	14	28	36.640 ± 0.208

Table S5. Factor levels in Box-Behnken design.

Factor	Variable	Level		
		-1	0	+1
Glucose (g/L)	B	65	75	85
Peptone (g/L)	C	10	11	12
Yeast extract (g/L)	D	20	22	24

Table S6. Experimental design and corresponding fungal biomass in Box-Behnken design.

Run	Glucose (g/L)	Peptone (g/L)	Yest extract (g/L)	Biomass (g/L)
1	65	11	24	38.640 ± 0.219
2	75	11	22	45.220 ± 0.266
3	85	11	20	38.700 ± 0.219
4	65	10	22	36.640 ± 0.218
5	85	12	22	38.840 ± 0.219
6	85	11	24	39.200 ± 0.235
7	65	12	22	39.100 ± 0.219
8	75	11	22	43.720 ± 0.256
9	75	11	22	45.680 ± 0.266
10	85	10	22	40.160 ± 0.231
11	75	10	24	38.440 ± 0.219
12	65	11	20	35.520 ± 0.208
13	75	10	20	37.400 ± 0.219
14	75	12	20	38.280 ± 0.219
15	75	11	22	43.800 ± 0.254
16	75	12	24	39.720 ± 0.231
17	75	11	22	45.060 ± 0.266

Table S7. Concentration and volume of salt solutions for electrolyte stock solutions of different simulated digestion fluids.

Salt solution (M)	Volume (mL)		
	SSF (pH 7)	SGF (pH 3)	SIF (pH 7)
KCl (0.5)	3.8	1.725	1.7
KH_2PO_4 (0.5)	0.925	0.225	0.2
$NaHCO_3$ (1)	1.7	3.125	10.625
NaCl (2)	-	2.95	2.4
$MgCl_2(H_2O)_6$ (0.15)	0.125	0.1	0.275
$(NH_4)_2CO_3$ (0.5)	0.015	0.125	-

SSF, simulated saliva digestion fluid;

SGF, simulated gastric digestion fluid;

SIF, simulated intestinal digestion fluid.

Table S8. Analysis of variance (ANOVA) for the quadratic model of the Box-Behnken design.

Source	Sum of Squares	df	Mean Square	F-value	*p*-value
Model	0.3972	9	0.0441	35.80	< 0.0001**
B	0.0153	1	0.0153	12.42	0.0097**
C	0.0034	1	0.0034	2.76	0.1406
D	0.0116	1	0.0116	9.43	0.0180*
BC	0.0089	1	0.0089	7.24	0.0310*
BD	0.0043	1	0.0043	3.48	0.1044
CD	0.0001	1	0.0001	0.081	0.7840
B^2	0.1097	1	0.1097	88.97	< 0.0001**
C^2	0.0815	1	0.0815	66.13	< 0.0001**
D^2	0.1255	1	0.1255	101.80	< 0.0001**
Residual	0.0086	7	0.0012	-	-
Lack of Fit	0.0008	3	0.0003	0.14	0.9330
Pure Error	0.0078	4	0.0020	-	-
Cor Total	0.4059	16	-	-	-

B: glucose.

C: peptone.

D: yeast extract.

*: Significance with $p < 0.05$.

**:Significance with $p < 0.01$.

Table S9. Reducing sugar content of soluble starch during *in vitro* digestion.

Digestion time (min)	Reducing sugar content (%, w/w)
	Soluble Starch
SSF	
0min	9.03 ± 0.05^{a}
5min	18.45 ± 0.11^{c}
SGF	
0min	13.24 ± 1.77^{b}
1h	12.05 ± 0.06^{b}
2h	11.92 ± 0.04^{b}
SIF	
0min	29.02 ± 0.17^{d}
1h	31.44 ± 0.16^{e}
2h	33.85 ± 0.11^{f}

SSF, simulated saliva digestion fluid;

SGF, simulated gastric digestion fluid;

SIF, simulated intestinal digestion fluid.

Table S10. Sobs and Shannon index of bacteria with interference of different polysaccharides.

Polysaccharides	Sobs index	Shannon index
Blank_1	144	2.69
Blank_2	124	2.39
Blank_3	133	2.54
Inulin_1	87	1.44
Inulin_2	92	1.47
Inulin_3	97	1.39
EPS_1	154	2.88
EPS_2	147	2.92
EPS_3	139	2.88
IPS_1	155	2.64
IPS_2	150	2.54
IPS_3	156	2.70

Table S11. Bacterial relative abundance at phylum level.

%	Blank	IPS	EPS	Inulin
Firmicutes	30.19 ± 7.27^{bc}	24.74 ± 1.74^{c}	33.09 ± 2.07^{b}	71.19 ± 0.26^{a}
Bacteroidota	4.33 ± 0.52^{c}	54.57 ± 1.71^{a}	45.08 ± 2.16^{b}	2.97 ± 0.24^{c}
Proteobacteria	44.23 ± 3.46^{a}	18.36 ± 0.46^{b}	19.24 ± 0.36^{b}	7.76 ± 1.26^{c}
Actinobacteriota	3.56 ± 1.07^{b}	0.64 ± 0.11^{c}	0.91 ± 0.12^{d}	17.94 ± 1.23^{a}
Desulfobacterota	13.18 ± 1.22^{a}	0.92 ± 0.03^{b}	0.96 ± 0.13^{b}	0.14 ± 0.01^{c}
Fusobacteriota	4.04 ± 3.59^{a}	0.73 ± 0.27^{b}	0.72 ± 0.16^{b}	-

a, b, c, d: different letters mean significant difference ($p<0.05$) among different groups, ANOVA, n=3.

Table S12. Bacterial relative abundance at genus level.

%	Blank	IPS	EPS	Inulin
Escherichia-Shigella	35.79 ± 2.32^{a}	14.22 ± 0.14^{b}	15.38 ± 0.15^{b}	5.54 ± 1.04^{c}
Megamonas	-	5.29 ± 0.63^{b}	3.23 ± 0.72^{c}	61.81 ± 0.77^{a}
Parabacteroides	0.54 ± 0.12^{c}	39.09 ± 4.31^{a}	28.35 ± 2.19^{b}	0.37 ± 0.03^{c}
Bacteroides	3.70 ± 0.46^{b}	15.39 ± 3.92^{a}	16.62 ± 0.94^{a}	2.18 ± 0.22^{c}
Phascolarctobacterium	6.48 ± 3.44^{a}	8.22 ± 1.37^{a}	9.33 ± 2.37^{a}	0.30 ± 0.04^{b}
Bifidobacterium	3.43 ± 1.06^{b}	0.45 ± 0.07^{c}	0.59 ± 0.09^{c}	17.89 ± 1.22^{a}
Lachnoclostridium	8.89 ± 1.98^{a}	3.59 ± 0.14^{b}	4.29 ± 0.29^{b}	-
Klebsiella	8.22 ± 1.17^{a}	2.08 ± 0.09^{b}	3.30 ± 0.54^{b}	2.22 ± 0.22^{b}
Lactobacillus	0.59 ± 0.19^{b}	-	-	7.73 ± 0.92^{a}
Desulfovibrio	7.08 ± 0.53^{a}	0.31 ± 0.01^{b}	0.35 ± 0.06^{b}	-
Bilophila	6.10 ± 0.80^{a}	0.62 ± 0.03^{b}	0.61 ± 0.08^{b}	0.14 ± 0.01^{b}
Lachnospiraceae_UCG-004	2.44 ± 3.43^{a}	1.93 ± 0.35^{a}	3.08 ± 0.15^{a}	-
Dialister	-	2.50 ± 0.65^{a}	3.18 ± 0.31^{a}	0.24 ± 0.02^{b}
Enterococcus	4.62 ± 2.59^{a}	0.20 ± 0.02^{b}	0.64 ± 0.12^{b}	0.18 ± 0.03^{b}

Table S12. *(Contd.)*

%	Blank	IPS	EPS	Inulin
Allisonella	1.56 ± 0.33[b]	1.60 ± 0.19[b]	2.34 ± 0.24[a]	-
Fusobacterium	4.04 ± 3.59[a]	0.73 ± 0.27[a]	0.72 ± 0.16[a]	-
Sutterella	-	1.73 ± 0.31[a]	0.37 ± 0.04[b]	-
Eubacterium	1.79 ± 0.19[a]	0.09 ± 0.02[b]	0.07 ± 0.01[b]	0.03 ± 0.01[b]
Faecalibacterium	0.08 ± 0.04[c]	0.51 ± 0.05[b]	0.78 ± 0.06[a]	0.10 ± 0.02[c]
Romboutsia	0.59 ± 0.37[a]	0.05 ± 0.01[b]	0.09 ± 0.01[b]	0.31 ± 0.06[a]
Clostridium_sensu_stricto_1	0.03 ± 0.03[b]	-	0.99 ± 0.25[a]	-
Dorea	0.05 ± 0.03[c]	0.41 ± 0.05[b]	0.57 ± 0.02[a]	-
Blautia	0.30 ± 0.010[b]	0.19 ± 0.03[c]	0.37 ± 0.03[a]	0.03 ± 0.01[d]
Subdoligranulum	0.39 ± 0.04[a]	0.17 ± 0.06[bc]	0.22 ± 0.03[b]	0.09 ± 0.02[c]
Parasutterella	0.18 ± 0.05[b]	0.31 ± 0.05[a]	0.18 ± 0.01[b]	-
Sellimonas	0.07 ± 0.03[c]	0.38 ± 0.03[a]	0.21 ± 0.02[b]	-
unclassified_f_Lachnospiraceae	0.53 ± 0.10[a]	0.07 ± 0.03[b]	0.04 ± 0.01[b]	0.01 ± 0.01[b]
Alistipes	0.07 ± 0.01[b]	0.07 ± 0.03[b]	0.08 ± 0.00[b]	0.35 ± 0.03[a]

Table S12. *(Contd.)*

%	Blank	IPS	EPS	Inulin
Ruminococcus_torques_group	0.34 ± 0.06^{a}	0.11 ± 0.02^{b}	0.08 ± 0.01^{b}	0.04 ± 0^{b}
Eubacterium_hallii_group	0.06 ± 0.03^{c}	0.19 ± 0.03^{b}	0.26 ± 0.03^{a}	-
others	1.95 ± 0.17^{a}	1.54 ± 0.13^{b}	1.57 ± 0.11^{b}	0.44 ± 0.01^{c}

a, b, c, d: different letters mean significant difference ($p<0.05$) among different groups, ANOVA, n=3.

Table S13. Relative abundance of several beneficial metabolites.

%	Blank	IPS	EPS
Chenodeoxycholylarginine	3.87 ± 0.79^{c}	5.80 ± 0.04^{b}	6.03 ± 0.03^{a}
25-Hydroxyvitamin D2	3.17 ± 0.01^{b}	3.18 ± 0.01^{b}	5.29 ± 0.13^{a}
Guanine	3.02 ± 0.01^{b}	3.03 ± 0.01^{b}	3.82 ± 0.07^{a}
Spermidine	6.10 ± 0.02^{c}	6.60 ± 0.05^{a}	6.32 ± 0.09^{b}
Gallic acid	2.43 ± 0.58^{b}	5.57 ± 0.10^{a}	5.65 ± 0.09^{a}
3-Indolepropionic acid	4.24 ± 0.60^{b}	5.58 ± 0.01^{a}	5.56 ± 0.05^{a}

a,b,c: different letters mean significant difference ($p<0.05$) among different groups, ANOVA, n=3.
